# SF3B1 inhibition disrupts malignancy and prolongs survival in glioblastoma patients through *BCL2L1* splicing and mTOR/ß-catenin pathways imbalances

**DOI:** 10.1186/s13046-022-02241-4

**Published:** 2022-01-27

**Authors:** Antonio C. Fuentes-Fayos, Jesús M. Pérez-Gómez, Miguel E. G-García, Juan M. Jiménez-Vacas, Cristóbal Blanco-Acevedo, Rafael Sánchez-Sánchez, Juan Solivera, Joshua J. Breunig, Manuel D. Gahete, Justo P. Castaño, Raúl M. Luque

**Affiliations:** 1 Maimonides Biomedical Research Institute of Cordoba (IMIBIC), 14004 Córdoba, Spain; 2 Department of Cell Biology, Physiology and Immunology, University of Cordoba, 14004 Cordoba, Spain; 3 Reina Sofia University Hospital (HURS), 14004 Cordoba, Spain; 4 CIBER Physiopathology of Obesity and Nutrition (CIBERobn), 14004 Cordoba, Spain; 5 Department of Neurosurgery, Reina Sofia University Hospital, 14004 Cordoba, Spain; 6 Pathology Service, Reina Sofia University Hospital, 14004 Cordoba, Spain; 7 Board of Governors Regenerative Medicine Institute, Cedars-Sinai Medical Center, Los Angeles, CA 90048 USA; 8 Center for Neural Sciences in Medicine, Cedars-Sinai Medical Center, Los Angeles, CA 90048 USA; 9 Department of Biomedical Sciences, Cedars-Sinai Medical Center, Los Angeles, CA 90048 USA; 10 Samuel Oschin Comprehensive Cancer Institute, Cedars-Sinai Medical Center, Los Angeles, CA 90048 USA; 11 Department of Medicine, David Geffen School of Medicine, University of California, Los Angeles, CA 90095 USA

**Keywords:** Glioblastoma, Splicing factor SF3B1, Glioma mouse models, Antitumor therapy, *BCL2L1* splicing variants

## Abstract

**Background:**

Glioblastoma is one of the most devastating cancer worldwide based on its locally aggressive behavior and because it cannot be cured by current therapies. Defects in alternative splicing process are frequent in cancer. Recently, we demonstrated that dysregulation of the spliceosome is directly associated with glioma development, progression, and aggressiveness.

**Methods:**

Different human cohorts and a dataset from different glioma mouse models were analyzed to determine the mutation frequency as well as the gene and protein expression levels between tumor and control samples of the splicing-factor-3B-subunit-1 (SF3B1), an essential and druggable spliceosome component. *SF3B1* expression was also explored at the single-cell level across all cell subpopulations and transcriptomic programs. The association of *SF3B1* expression with relevant clinical data (e.g., overall survival) in different human cohorts was also analyzed. Different functional (proliferation/migration/tumorspheres and colonies formation/VEGF secretion/apoptosis) and mechanistic (gene expression/signaling pathways) assays were performed in three different glioblastomas cell models (human primary cultures and cell lines) in response to SF3B1 blockade (using pladienolide B treatment). Moreover, tumor progression and formation were monitored in response to SF3B1 blockade in two preclinical xenograft glioblastoma mouse models.

**Results:**

Our data provide novel evidence demonstrating that the splicing-factor-3B-subunit-1 (SF3B1, an essential and druggable spliceosome component) is low-frequency mutated in human gliomas (~ 1 %) but widely overexpressed in glioblastoma compared with control samples from the different human cohorts and mouse models included in the present study, wherein SF3B1 levels are associated with key molecular and clinical features (e.g., overall survival, poor prognosis and/or drug resistance). Remarkably, in vitro and in vivo blockade of SF3B1 activity with pladienolide B drastically altered multiple glioblastoma pathophysiological processes (i.e., reduction in proliferation, migration, tumorspheres formation, VEGF secretion, tumor initiation and increased apoptosis) likely by suppressing AKT/mTOR/ß-catenin pathways, and an imbalance of *BCL2L1* splicing.

**Conclusions:**

Together, we highlight SF3B1 as a potential diagnostic and prognostic biomarker and an efficient pharmacological target in glioblastoma, offering a clinically relevant opportunity worth to be explored in humans.

**Supplementary Information:**

The online version contains supplementary material available at 10.1186/s13046-022-02241-4.

## Background

Gliomas are the most frequent (> 80%) primary malignant brain tumor in adults [55]. They are classified into low-grade (I and II) and high-grade (III and IV) gliomas based on integrated classic histological/molecular features [43]. Grade IV astrocytoma, the most prevalent glioma, known as glioblastoma multiforme (GBM), is one of the most devastating and malignant cancers [55] and its incidence has increased relevantly in recent years, while in other gliomas remained stable [57]. Despite significant advances in the knowledge of GBM pathophysiology, it remains an incurable disease with median survival after diagnosis of ~ 15 months [54, 61]. Still, effective therapeutic targets are severely lacking, and, therefore, innovative therapeutic approaches are urgently needed [62].

Growing evidence indicates that defects in the alternative splicing process are frequent in cancer, which has gained important attention in the past 10 years [27, 53]. Moreover, our group and others have demonstrated that the spliceosome, the cellular machinery controlling the splicing process, is drastically altered in GBM and different cancer types [7, 23, 31, 34, 67], leading to the appearance of aberrant/oncogenic splicing variants (SVs) from different genes [e.g., *GFAP* [47]*/VEGF* [26]*/TP53* [2]*/BCL2L1* [72]*/TP73* [23]]. Specifically, we have demonstrated that the dysregulation of the spliceosome is associated with GBM development/progression/aggressiveness, which could potentially be considered as a source of novel diagnostic/prognostic-biomarkers and therapeutic targets to combat this devastating pathology [23].

The splicing-factor-3B-subunit-1 (SF3B1) is a core spliceosome component essential for splicing function [66]. SF3B1 gained importance due to many functionally deleterious mutations found in various cancer types [41] [i.e., myelodysplastic syndrome [29]/breast cancer [20]/prolactinomas [38]/uveal melanoma [32]/pancreatic ductal adenocarcinoma [4]], which are associated with patient poor-prognosis/survival. Additionally, we have recently found that *SF3B1* is overexpressed and associated with malignant features in prostate cancer [30] and hepatocellular carcinoma [42], supporting that SF3B1 could represent a valuable therapeutic target in cancer. Accordingly, various drugs have now been designed to specifically target SF3B1, including pladienolide B, a selective inhibitor that disrupts the spliceosome assembly [16, 35, 48]. However, to the best of our knowledge, the oncogenic implication of SF3B1, its somatic mutations, and expression profile or its association with molecular features and clinical parameters have not been characterized in GBM, nor its putative therapeutic potential. Therefore, different human cohorts and a dataset from different glioma mouse models were analyzed to determine the mutation frequency as well as the gene and protein expression levels between tumor and control samples of the SF3B1, an essential and druggable spliceosome component. *SF3B1* expression was also explored at the single-cell level across all cell subpopulations and transcriptomic programs. The association of *SF3B1* expression with relevant clinical data (e.g., overall survival) in different human cohorts was also analyzed. Moreover, several functional and molecular endpoints were measured in different GBM cell models (human primary cultures and two cell lines) after SF3B1 blockade (using pladienolide B treatment). In addition, tumor progression and initiation in response to SF3B1 blockade were examined in two GBM xenograft mouse models. These analyses unveil SF3B1 as a potential biomarker being a novel pharmacological target in this devastating tumor.

## Methods

### Reagents

Unless otherwise indicated, reagents and products were purchased from Sigma-Aldrich. Pladienolide B was obtained from Santa-Cruz Biotechnology (CAS 445493–23-2).

### Mutation analysis in glioma samples

CGGA [WEseq data [73]; *n* = 284], TCGA [WEseq data [11]; *n* = 746] and MSKCC [DNAseq data [33]; *n* = 923] were interrogated through the cBioportal website (www.cbioportal.org) and OncoPrinter tool (www.cbioportal.org/oncoprinter) (Table S1).

### Patients and samples

Fresh GBM samples (*n* = 22) were obtained by intracranial surgery and non-tumor samples from 4 healthy brain donors by autopsy (Table S2). All samples were histologically confirmed by expert anatomic pathologists. Samples were cut and rapidly frozen in liquid nitrogen and then stored at − 80 °C until extraction for total-RNA or formalin-fixed paraffin-embedded (FFPE) immunohistochemical (IHC)-analysis (see below). Demographic and clinical characteristics were collected to perform clinical correlations. This study was approved by Reina Sofia University Hospital Ethics Committee and was conducted by the principles of the Helsinki Declaration. Written informed consent was obtained from all individuals.

### Bioinformatic analysis of in silico cohorts for RNAseq and proteomic data

All the bioinformatic methodology was implemented in R language 3.5. Specifically: i) Rembrandt microarray (*n* = 219 GBM; *n* = 28 non-tumor) and CGGA (bulk-RNAseq data; *n* = 388) were interrogated through the GlioVis-Tools (http://gliovis.bioinfo.cnio.es) (Table S2). ii) Single-cell RNAseq data of adult GBM were downloaded from Single-cell -Portal – Broad-Institute (GSE131928; total adult cells, *n* = 5528) [50] and analyzed using Seurat-packageV3 [60] . Filtering was performed removing cells with < 200 and > 8000 features and selecting cells with a percentage of mitochondrial genes over 0.9 (*n* = 5123 filtered cells were obtained; Fig. S1a-b). Data were normalized using LogNormalize-method and scaled with a factor = 10,000. PCA and UMAP methods were applied to perform cell clustering (Fig. S1c-d). Top 10 markers were used to characterize each cluster (Table S3/Fig. S1e). Transcriptional programs were classified using a relative meta-module score [log2(|SC1-SC2| + 1)] [50]. iii) Paired-end bulk-RNAseq data from EPed mouse models have been aligned against UCSC hg19 assembly using STAR2.7.0a. Normalization, count per gene associations, and differential expression analysis were achieved by Partek Flow® software (Partek Incorporated, St. Louis, MO, USA). iv) CPTAC GBM Discovery Study proteomic data (*n* = 100 GBM; *n* = 10 Non-tumor; Table S4) were downloaded from https://cptac-data-portal.georgetown.edu [19]. v) Genomics of Drug Sensitivity in Cancer (GDSC) database was used to determine the resistance and sensitivity of 100 compounds on 18 GBM cell lines (https://www.cancerrxgene.org) and combined with *SF3B1* expression of GBM cell lines from Broad Institute Cancer Cell Line Encyclopedia (CCLE-https://portals.broadinstitute.org/ccle). vi) Group of patients for survival analyses were selected based on the cutoff points determined by *survminer R package.* vii) STRING database (https://string-db.org) was used to determine the potential functional association between several genes correlated with *SF3B1* (r > ± 0.800). Enrichment analysis was performed based on KEGG-Pathways Analysis (Table S5). Reactome database was used to identify relevant pathways associated with *SF3B1 *expression and plotted using *ggplot2 R package*.

### Electroporated (EPed) mouse models

All experimental procedures were performed according to the Cedars-Sinai Institutional Animal-Care and Use Committee. Paired-end bulk-RNAseq datasets from previously generated glioma mouse models [with constitutively active oncogenes (Erbb2-V664E-EGFP/Hras-G12-EGFP/Kras-G12V-EGFP)] were used as previously described [9, 23].

### RNA isolation, quantitative real-time RT-PCR (qPCR), and customized qPCR dynamic array based on microfluidic technology

Total RNA from fresh non-tumor and tumor human samples and from GBM cell lines was extracted and DNase-treated, the concentration quantified, and the RNA retro-transcribed for qPCR analyses as previously described [9, 23]. As recently reported [30, 31], qPCR dynamic array based on microfluidic technology was implemented to determine the expression of *SF3B1* simultaneously in human samples and cell lines. Specific primers for human transcripts including *SF3B1*, key GBM tumor markers, selected signaling pathway endpoints genes and 3 housekeeping genes were specifically designed with the Primer3 4.0.0 software (Table S6). To control for variations in the efficiency of the retrotranscription -reaction, mRNA copy numbers of the different transcripts analyzed were adjusted by a normalization factor, calculated with the expression levels of 3 housekeeping genes [β-actin (*ACTB*), hypoxanthine-guanine phosphoribosyl-transferase (*HPRT*), glyceraldehyde 3-phosphate dehydrogenase (*GAPDH*); Table S6] and the GeNorm 3.3 software as previously reported [31, 44].

### Immunohistochemical (IHC) analysis

IHC of SF3B1 was performed on FFPE samples obtained by intracranial surgery from patients diagnosed with GBM (*n* = 13) and control/non-pathologic samples (*n* = 4) from our cohort (Table S2). Specifically, rabbit polyclonal-antibodies against human SF3B1 (Abcam, #ab172634) were used following the manufacturer’s instructions. Specifically, deparaffinized sections were incubated with the antibody overnight at 4 °C. Then, ImmPRESS® Anti-Mouse/Rabbit IgG PEROXIDASE (Vector-Laboratories, #MP-7500-50) was used according to the supplier’s recommendations. Finally, sections were developed with 3,39-diaminobenzidine (EnvisionSystem 2-KiTSolution-DAB, ThermoFisher-Scientific, #34065), contrasted with hematoxylin (#MHS128). As previously reported [17, 31], the pathologists performed histopathologic analyses of the samples following a blinded protocol. In the analysis, 1(+), 2(++), 3(+++) indicate low, moderate, and high intensities of tumor-region staining compared with the non-tumor/ control adjacent region.

### GBM cell lines

U-87 MG and U-118 MG cells were obtained from the American Type Culture Collection (ATCC, #HTB-14/ #HTB-15, respectively) and cultured according to the supplier’s recommendations. These cell lines were previously checked for mycoplasma contamination by PCR as previously reported [65].

### Primary patient-derived GBM and non-tumor brain cell cultures

Fresh tissue samples were collected within 15 min after intracranial surgery and immediately transported to the cell culture room in sterile cold S-MEM medium (Gibco, #11380–037) complemented with 0.1% BSA (#A2153), 0.01% L-glutamine (#G7513), 1% antibiotic-antimycotic solution (Gibco, #R01510) and 2.5% HEPES (#H3537). Fresh tissue samples were dispersed into single-cells within the following 30 min by a mechanic/enzymatic protocol as previously reported [23]. The single-cells were cultured onto coating poly-L-Lysine (#P1524-25MG) tissue-culture plates in a 10% FBS (#F6765) containing D-MEM (BI, #06–1055-09-1A) complemented as an S-MEM medium. All the GBM processed were *IDH1*wt.

### Dose-response, IC_50_ determination, and measurements of proliferation and migration rates

Proliferation assay was used to perform a dose-response [1 nM, 100 nM, and 10 μM; dose selected based on previously reported in vitro studies [30, 67]] and IC_50_ determination (at 48 h) of pladienolide B in GBM cell lines and primary-GBM cell cultures. Least-squares regression was used as a fitting method to IC_50_ determination. As previously described [28], cell proliferation was analyzed using alamarBlue™ assay (5,000 cells/well for cell lines and 10,000 cells/well for primary cell-cultures; Biosource International, #BUF012B), and migration using the wound-healing technique (150,000 U-118 MG cells/well). For the migration assay, U-118 MG cells cultured under confluence were serum-starved for 24 h to achieve cell synchronization, and then, the wound was made using a 200 μl sterile pipette tip. Wells were replaced and cells were incubated for 6 h and 24 h with supplemented medium without FBS. Wound-healing was compared with the area just after the wound was performed. Three pictures were randomly acquired along the wound per well to calculate the area by ImageJ 1.8.0_172 software [58].

### Apoptosis measurement

Apoptosis induction in response to pladienolide B treatment in GBM cell lines (5,000 cells/well onto white-walled multiwell luminometer plates) was performed by using Caspase-Glo® 3/7 Assay (Promega Corporation, #G8091) as previously reported [23]. In addition, Cleaved-Caspase 3 protein level was identified by western blot (see below) after pladienolide B treatment.

### Tumorspheres formation

Previously described assay [23] was carried out with both GBM cell lines (100 cells/well) cultured in a Corning Costar ultra-low attachment plate (#CLS3473) using D-MEM F-12 (Gibco, #11320033) with EGF (20 ng/μl) (#SRP3027) for 10 days (refreshing every 48 h, EGF and pladienolide B treatment) [23]. Additionally, tumorspheres formation was measured in U-87 MG and U-118 MG cells, pre-treated with pladienolide B (24 h and 48 h) before seeding the experiment. Photographs were taken to visualize and measure the area after 10 days of incubation with pladienolide B.

### VEGF secretion

The VEGF Human ELISA Kit (ThermoFisher-Scientific, #KHG0112) was used to quantify VEGF secretion in response to pladienolide B in GBM cell lines, following the manufacturer’s instructions and previously described methods [23].

### Colony formation

Colony formation assay was performed in GBM cell lines. Briefly, 300 or 500 cells/well (6-well plate) of U-87 MG and U-118 MG were seeded, respectively. Cells were pre-treated with pladienolide B (for 24 h and 48 h) before seeding the experiment to evaluate its effect on tumor onset/formation. Then, cells were seeded, medium was replaced, cells washed with PBS 1x, and crystal violet 0.5% plus glutaraldehyde 6% was added and incubated 45 min at room temperature. Finally, cells were rinsed 3 times with distilled water and left to dry at room temperature. Colonies (particles per well) were measured by ChemiDoc-XRS+ System (Bio-Rad, Hercules, CA) and analyzed using ImageJ 1.8.0_172 software.

### Western blotting

To determine protein levels, cell pellets were resuspended using pre-warmed SDS-DTT sample buffer [62.5 mM Tris-HCl (#10708976001), 2% SDS (#71726), 20% glycerol (#17904), 100 mM DTT (#D0632-5G) and 0.005% bromophenol-blue (#B0126)] followed by sonication for 10 s and boiling for 5 min at 95 °C. Proteins were separated by SDS-PAGE electrophoresis with different poly-acrylamide percentage and transferred to nitrocellulose-membranes (Millipore, #1704270). Membranes were blocked with 5% non-fat dry milk (#T145.3) in Tris-buffered saline/0.05%-Tween-20 (#93773) and incubated with the primary-antibody [SF3B1 (Abcam, #ab172634), Cleaved-Caspase 3 (CST, #9664), phospho-AKT (CST, #4060), AKT (CST, #9272), phospho-MTOR (CST, #2971), MTOR (CST, #2972), phospho-p70-S6K1 (CST, #9205), p70-S6K1 (CST, #9202), phospho-PDK1 (CST, #3061), phospho-TP53 (SCBT, #sc-135772), TP53 (Cusabio, #CSB-MA0240771A0m), HIF1A (Novus Biological, #NB100–134), ACTB (Sigma, #A5441), TUBB (Abcam, #ab6046) and CTNNB1 (CST, #8480)], and their appropriate secondary-antibodies [anti-rabbit (CST, #7074) or anti-mouse (CST, #7076)]. Proteins were detected using an enhanced chemiluminescence-detection system (GE-Healthcare) with dyed molecular-weight markers. As previously reported [31], a densitometric analysis of the bands was carried out with ImageJ 1.8.0_172 software [58] using total-protein loading (Ponceau-staining, #P3504-10G) or total-protein signal (in case of AKT and ERK) as normalizing factor, and represented using a heatmap and box-plots. The suitability of Ponceau staining as internal control compared with ACTB or TUBB was confirmed (Fig. S1g).

### Preclinical mouse models, Micro-CT imaging, and Hematoxylin & Eosin examination

A preclinical xenograft mouse model to test pladienolide B in vivo was developed. 5-week-old ATHYM-*Foxn1*^nu/nu^ mice (*n* = 6; Janvier-Labs) were injected subcutaneously with 3 × 10^6^ of U-87 MG cells in both flanks [resuspended in 100 μl of basement membrane extract (Trevigen, #3432–010-01)]. Once the tumor was clearly measurable, each mouse received an intra-tumor injection (12 days after cell-inoculation) with 50 μl of pladienolide B (100 nM) into one flank and vehicle (1xDulbecco’s Phosphate-Buffered Saline, Sigma-Aldrich, #D1408; used as control) into the other flank. Tumor growth was monitored every 2 days using a digital caliper. Eight days after injection, mice were sacrificed and each tumor was dissected, fixed, and sectioned for histopathologic examination after H&E-staining. Examination of mitosis number, vascular proliferation, and necrosis was performed by expert pathologists. Additional tumor pieces were placed in liquid nitrogen and then frozen at -80 °C until RNA or protein extraction using Trizol-reagent or SDS-DTT buffer, respectively, and as previously reported [23]. Micro-CT imaging using SkyScan1176 Bruker and software environment was used to show in vivo tumor location previous to dissection. Specifically, 2D analysis together with 3D imaging rendering was performed using VolView 3.4 software (KitWare Inc).

In addition, a preclinical xenograft mouse model was developed by inoculating U-87 MG cells previously pre-treated with pladienolide B at 100 nM in vitro for 24 h and 48 h. Specifically, 5-week-old ATHYM-*Foxn1*^nu/nu^ mice were injected subcutaneously with 3 × 10^6^ of U-87 MG pre-treated cells [*n* = 6 mice/condition (i.e., cells pre-treated for 24 h or 48 h with pladienolide B in one flank and their corresponding vehicle-treated controls in the other flank)] using similar approaches described above. In this case, Slicer 4.11 software was used for 2D analysis together with 3D imaging rendering in the Micro-CT imaging. These experiments were performed according to the European Regulations for Animal Care under the approval of the university/regional-government research ethics committees.

### SVs detection by end-point-PCR and by qPCR in response to pladienolide B treatment

End-point-PCR was developed using cDNA from GBM cell lines, primary-GBM cell cultures, and U-87 MG xenograft mouse models in response to pladienolide B vs. control condition to detect SVs of *KLF6, CRK, MST1R, CASP2, RAC1, MCL1, BIRC5, SPP1,* and *BCL2L1* using specific primer pairs of each gene*.* Specifically, primer design for *BCL2L1* was performed using primers with specific annealing in *ExonIIa* (forward-sequence) and *ExonIII* (reverse-sequence) neighboring the splicing event (Fig. 9g). Different sized amplicons were estimated and subsequently identified by agarose gel-electrophoresis (*BCL2L1-xS*: *305 pb*; *BCL2L1-xL*: *494 pb*). Details of the end-point PCR to detect splicing events have been previously reported [18]. Then, qPCR was performed using the same cDNA samples using specific primers for each SVs (*BCL2L1-xS* and *BCL2L1-xL*) to quantify individually both SVs and calculate the ratio *BCL2L1-xS/BCL2L1-xL*. All primer sequences are included in Table S6.

### Antisense oligonucleotides design, transfection, and proliferation assay

Five different antisense oligonucleotides (ASOs; 18–24 bases) were designed based on different studies reporting how an ASO could be accurately designed to be stable inside the cell and identifying the most appropriated *BCL2L1* region [5, 24, 40, 45]. Briefly, these ASOs target different sequences of the ISS (Intron Splicing Silencer) region in the interexon region of* Bcl-xL/Bcl-xS exon II *and *III*, where the splicing is carried out. Phosphorothioate bonds binding all bases and four 5′ ends modified by 2′ O-methoxy-ethyl (2’MOE) residues were placed at the oligo’s end. Then, 100 nmol DNA Oligo was synthesized by Integrated DNA Technologies, Inc. [5, 45]. The ASO sequences are included in Table S7. For the cell proliferation assay, *ASO1_BCL2L1* and *ASO3_BCL2L1* were used. Briefly, 200,000 cells were transfected with 100 nM of each ASO individually using Lipofectamine™ 2000 (ThermoFisher-Scientific, #11668019) according to the manufacturer’s instructions. Nuclease-free water was used as a control condition. After 48 h, cells were collected for validation of the transfection (SVs detection) and seeded for proliferation assays (see above). Pladienolide B was administered 24 h before SV detection.

### Statistics

Data were evaluated for heterogeneity of variance by using the Kolmogorov–Smirnov test. Statistical differences were assessed by T-test, Mann–Whitney U test, or by 1-way ANOVA followed by Fisher’s correction exact test. Correlations were studied by using the Pearson correlation test. All statistical analyses were performed using Prism software 8.0 (GraphPad Software, La Jolla, CA, USA). *P*-value < 0.05 were considered statistically significant. Data represent median (interquartile-range) or means±SEM. Plus symbol (+) indicates a tendency between conditions (+*P* > 0.05 < 0.1). Asterisks (*P < 0.05; **P < 0.01; ***P < 0.001) indicate statistically significant differences, and “ns” indicates not statistically significant differences, across different conditions.

### Data availability

External bulk-RNAseq data analyzed in the present study are available in GlioVis-Tools (http://gliovis.bioinfo.cnio.es) and single-cell RNAseq data in the single-cell Portal-Broad Institute (https://singlecell.broadinstitute.org). The datasets generated and/or analyzed during the current study are available from the corresponding author upon reasonable request.

## Results

### *SF3B1* is mutated in gliomas

**Fig. 1:**
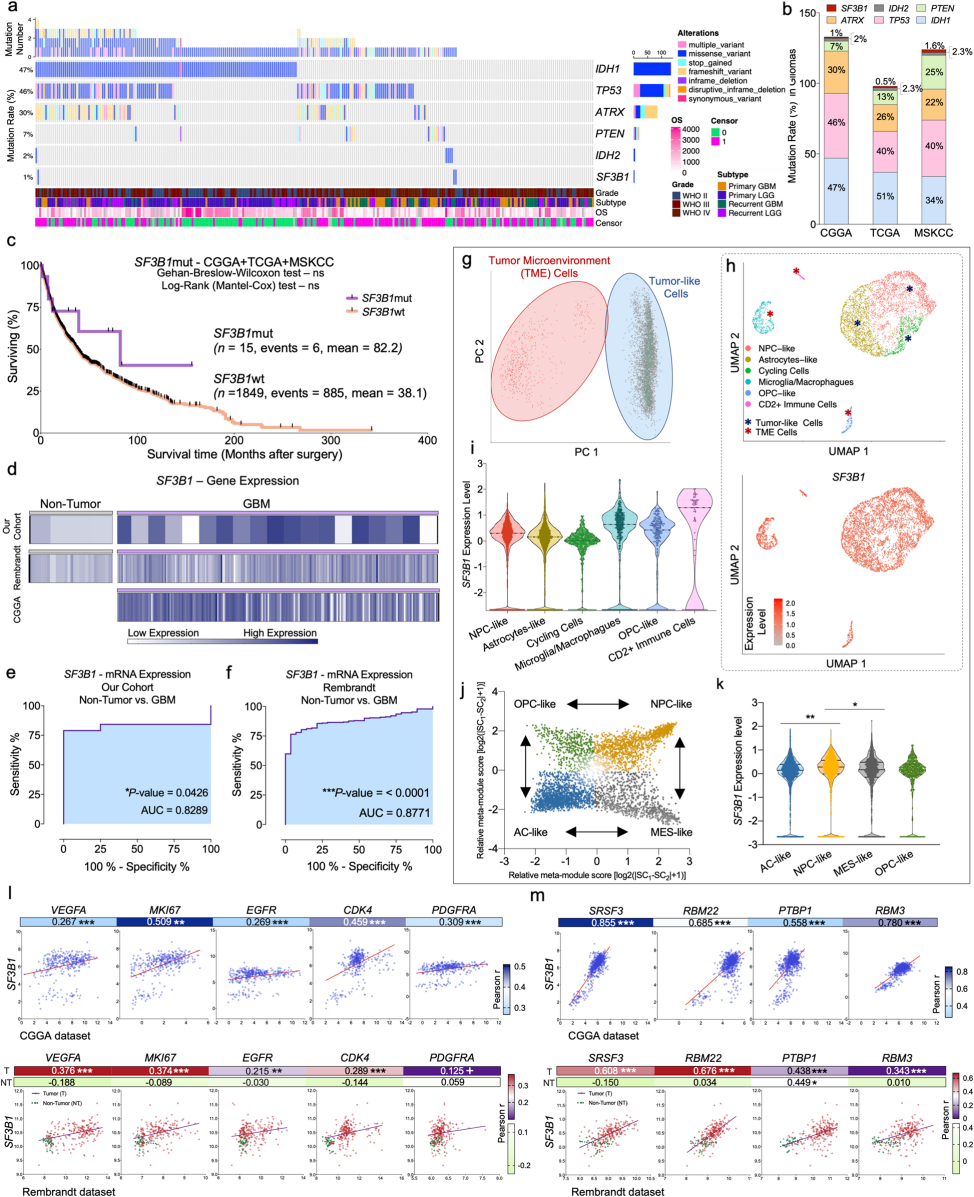
*SF3B1* is mutated and markedly overexpressed in human GBM samples compared to non-tumor brain samples. **a** Somatic mutation rate of *SF3B1* as well as of commonly mutated genes in glioma samples (*IDH1, TP53, ATRX, PTEN, IDH2*) obtained from the CGGA-dataset (*n* = 284 patients). Type of alterations, Overall Survival (OS), censored samples, glioma grade, and subtype are also indicated. **b** Percentage and similarity of somatic mutations rate of *IDH1, TP53, ATRX, PTEN, IDH2* and *SF3B1* genes in gliomas across the three different available datasets [CGGA-dataset (*n* = 284); TCGA-dataset (*n* = 746); MSKCC-dataset (*n* = 923)]. **c** Kaplan-Meier survival curves for glioma patients with mutated and wildtype *SF3B1* obtained from the CGGA-, TCGA- and MSKCC-datasets (*SF3B1*mut, *n* = 15; *SF3B1*wt, *n* = 1849). **d** Non-hierarchical heatmap generated comparing the expression levels of *SF3B1* in control brain tissues and/or GBM samples using our cohort, Rembrandt, and CGGA cohorts. Receiver-Operating-Characteristic (ROC)-curve analysis of *SF3B1* expression using control and GBM samples from our cohort (**e**) and the external Rembrandt cohort (**f**). Single-cell characterization of *SF3B1* through intra-tumor human cell populations: [**g** Principal components analysis (PCA) discriminating tumor microenvironment (TME) cells from tumor-like cells from a single-cell dataset. **h** Distribution of *SF3B1* expression in distinctive Uniform Manifold Approximation and Projection (UMAP) cluster (*Top panel:* Match of UMAP clusters with intra-tumor cell subtypes; *Bottom panel:* UMAP feature plot showing *SF3B1* expression). **i** *SF3B1* expression across different intra-tumor cell subtypes identified. **j** GBM cells classified by transcriptional programs in two-dimensional representation using Relative meta-module score [log2(|SC1-SC2| + 1)]. Each quadrant corresponds to one cellular state. **k** *SF3B1* expression across different GBM cell transcriptional programs]. Correlation of *SF3B1* with different key prognostic biomarkers (**l**) and relevant spliceosome components (**m**) in GBM samples from CGGA (upper panel) and Rembrandt (lower panel) cohorts including non-tumor samples for Rembrandt dataset. Asterisks (*P < 0.05; **P < 0.01; ***P < 0.001) indicate statistically significant differences across different conditions. Plus symbol (+) indicates a tendency between conditions (+*P* > 0.05 < 0.1)

*SF3B1* somatic-mutations (*SF3B1*mut) previously associated to alter physiological protein function [38, 49, 63] were characterized in 1953 glioma samples from 3 datasets (CGGA-dataset; TCGA-dataset; MSKCC-dataset) (Table S1). Specifically, we used these datasets to analyze *SF3B1*mut together with other classical mutated genes [*IDH1/TP53/ATRX/PTEN*/*IDH2;* Fig. 1a (CGGA-dataset); Fig. S2a (TCGA-dataset); Fig. S2b (MSKCC-dataset)]. These analyses revealed that *SF3B1*mut was observed in ~ 1% of patients (1%-CGGA; 0.5%-TCGA and 1.6%-MSKCC; Fig. 1a; Fig. S2a-b), being its frequency lower than those of the other classically mutated genes [i.e., *IDH1* (44%), *TP53* (42%), *ATRX* (26%), *PTEN* (15%), and *IDH2* (2%); Fig. 1b]. Moreover, no difference was observed in mean overall survival (OS) between *SF3B1*mut vs. *SF3B1*wt patients when all the cohorts/datasets were analyzed together (Fig. 1c) or individually (Fig. S2c-e).

### *SF3B1* is markedly overexpressed in human GBM samples compared to non-tumor brain samples

*SF3B1* mRNA levels were analyzed in three different human cohorts (Table S2). Specifically, a marked *SF3B1* overexpression was found in GBM compared to non-tumor brain tissues (control-tissues) in our cohort (*n* = 22 and 4, respectively; Fig. 1d; Fig. S2f), which was also corroborated in another well-characterized external patient cohort (Rembrandt; *n* = 219 and 28, respectively; Fig. 1d; Fig. S2g). Moreover, we also observed that the expression levels of *SF3B1* found in the tumor samples of these two cohorts were comparable with those found in the CGGA database (*n* = 388 GBM-samples; control-samples are not available; Fig. 1d [8]. Notably, Receiver-Operating-Characteristic (ROC)-curve analyses revealed the capacity of *SF3B1* levels to strongly discriminate between GBM vs. control-tissues, showing an Area Under the Curve (AUC) of 0.83 (our cohort) and 0.88 (Rembrandt-dataset) (Fig. 1e-f, respectively).

### *SF3B1* single-cell characterization in human intra-tumor cell populations

*SF3B1* expression was analyzed at single-cell level (GSE131928; *n* = 5528), which includes tumor microenvironment (TME) and tumor-like cells (Fig. 1g). Clustering analysis and classification based on cellular markers uncovered three different TME cell populations (Microglia/Macrophages, CD2+ immune cells, and OPC-like cells) and three tumor-like cells (NPC-like cells, Astrocyte-like cells, and Cycling cells) (Fig. 1h top-panel, and Table S3), being all these cell populations directly associated with tumor progression and dissemination [25, 50]. *SF3B1* expression was homogeneously present across the different cell populations (Fig. 1h bottom-panel, and Fig. 1i), being this expression virtually higher in TME vs. tumor-like cells (Fig. 1i; Fig. S1f). Likewise, *SF3B1* was expressed in all transcriptional programs of GBM cells that recapitulate distinct neural cell states (NPC-like, MES-like, AC-like, OPC-like; Fig. 1j) [50], wherein a higher expression was found in neural progenitor-like program (with proliferative potential) than the other programs (Fig. 1k). Therefore, the ubiquitous expression of *SF3B1* across all intra-tumor cell types/states suggests that *SF3B1* might represent a potential and global pharmacological target against all GBM-populations.

### *SF3B1* expression is correlated with relevant oncogenic tumor markers in GBM samples

A strong association between *SF3B1* expression and key tumor -markers of development/progression (*VEGFA/MKI67/EGFR/CDK4*/*PDGFRA*) was found in GBM (CGGA- and Rembrandt-datasets), but not in the non-tumor samples (Rembrandt-dataset) (Fig. 1l). A robust correlation between *SF3B1* expression and the most critical oncogenic spliceosome components [*SRSF3/RBM22/PTPB1/RBM3* [23]] was also found in GBM (CGGA- and Rembrandt-datasets), but not in the non-tumor samples (with the exception of *PTBP1*; Rembrandt-datasets)(Fig. 1m). These data suggest a potential prognostic role of SF3B1 in GBM.

### *Sf3b1* overexpression is validated in electroporated (EPed)-glioma mouse -models

**Fig. 2:**
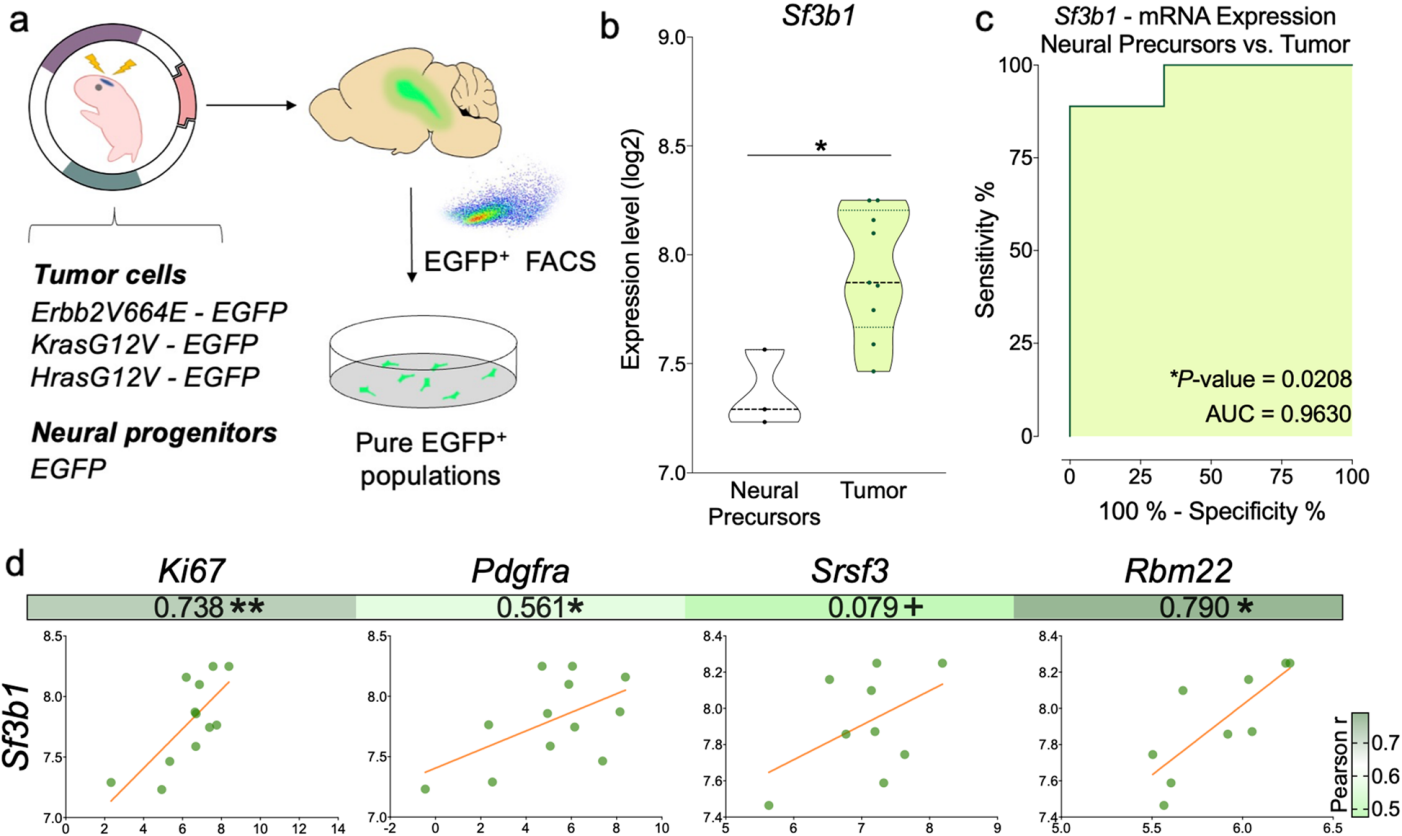
*Sf3b1* is overexpressed in different electroporated (EPed)-glioma mouse models. **a** Generation of mouse models of GBM by plasmid DNA mix injection into the left lateral ventricle following mouse brain electroporation (adapted from [9]). **b** mRNA expression levels of *Sf3b1* and, **c** ROC-curve analysis of *Sf3b1*, in the control and tumor samples of the EPed mouse model. **d** Correlation of *Sf3b1* with different key prognostic biomarkers and relevant spliceosome components in GBM samples from these models. Asterisks (*P < 0.05; **P < 0.01) indicate statistically significant differences across different conditions. Plus symbol (+) indicates a tendency between conditions (+*P* > 0.05 < 0.1)

*Sf3b1* overexpression was also corroborated in tumor samples from EPed mouse model vs. control samples from neural precursors [9] (Fig. 2a-b). ROC-curve analyses also supported the capacity of *Sf3b1* levels to discriminate between tumor vs. control samples, showing an AUC of 0.96 (Fig. 2c). Moreover, *Sf3b1* expression was also significantly correlated with key glioma/spliceosome -markers (*Mki67*/*Pdgfra/Rbm22*/*Sf3b1*; Fig. 2d).

### SF3B1 protein levels are elevated in GBM -samples

**Fig. 3:**
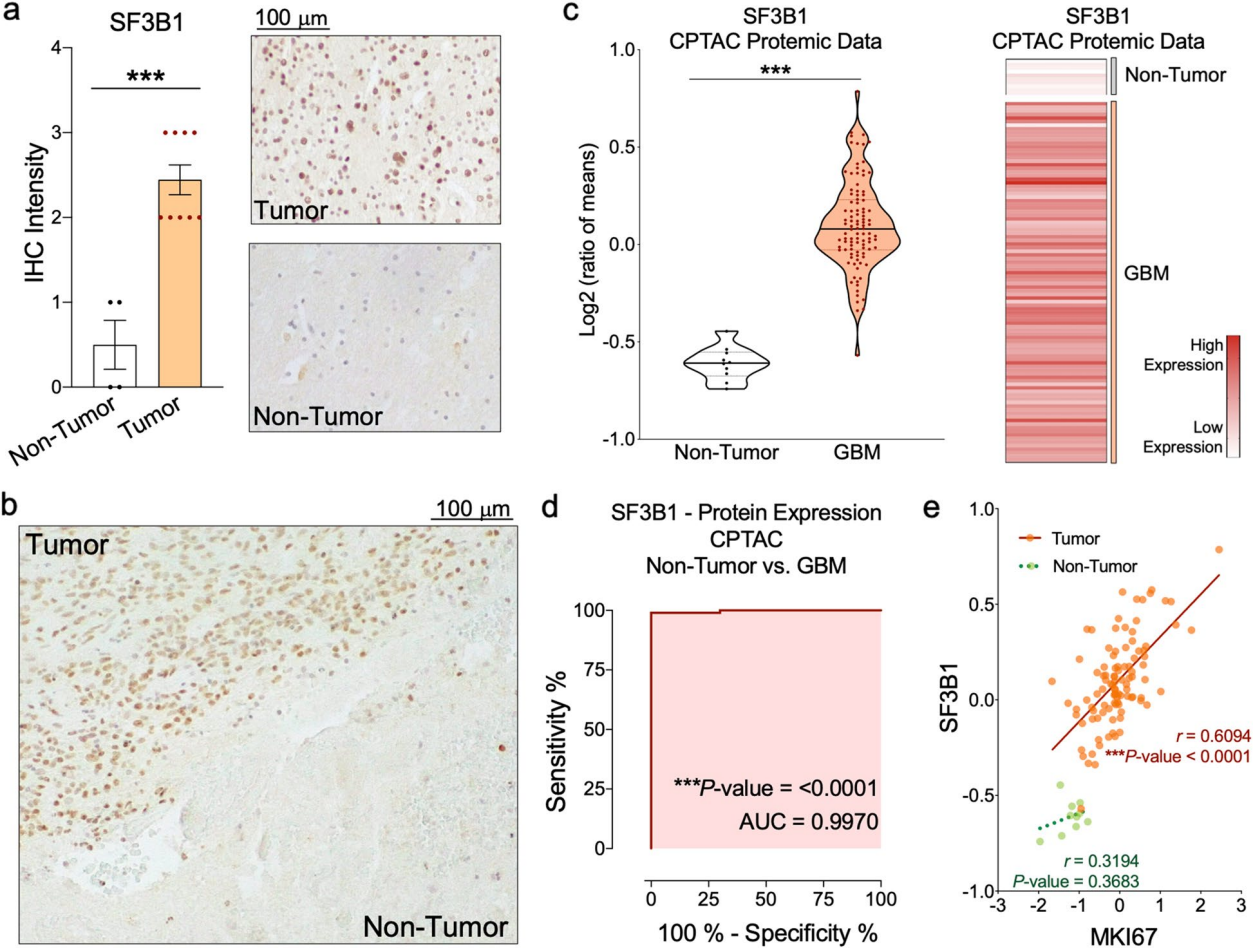
*SF3B1* is overexpressed at protein level in GBM samples. **a** Immunohistochemical (IHC) analysis of nuclear levels of SF3B1 in formalin-fixed paraffin-embedded (FFPE) samples from control and GBM tissues (representative images are shown). **b** IHC image comparing SF3B1 protein levels in an available GBM tissue vs. its non-tumor adjacent tissue. **c** SF3B1 protein levels in GBM [*Left panel:* SF3B1 protein levels compared to non-tumor samples (GTEx tissues) using the proteomic CPTAC dataset. *Right panel:* Non-hierarchical heatmap generated using the protein levels of SF3B1 in the same dataset]. **d** ROC-curve analysis of SF3B1 protein levels in the proteomic CPTAC dataset. **e** Correlation between protein levels of SF3B1 and the classical KI67 aggressiveness marker. Asterisks (***P < 0.001) indicate statistically significant differences across different conditions

Consistent with the mRNA results, IHC analyses of FFPE samples from our patient cohort (Table S2) revealed that nuclear SF3B1 protein levels were significantly elevated in GBM samples vs. non-tumor FFPE -samples (Fig. 3a). This drastic elevation was clearly observed in an available GBM -tissue vs. its non-tumor adjacent -tissue (Fig. 3b). Results were confirmed using CPTAC proteomic-data [19] (*n* = 100 GBM-samples vs. 10 control-tissues; Fig. 3c and Table S4). Moreover, ROC-curve analyses of SF3B1 protein levels confirmed its capacity to discriminate between GBM vs. control samples, showing an AUC of 0.99 (Fig. 3d). Additionally, we found a significant correlation between SF3B1 and MKI67 in GBM, but not in control tissues (Fig. 3e).

### *SF3B1* overexpression is associated with poor survival and prognostic in humans/mice and with drug-s resistance in GBM

**Fig. 4:**
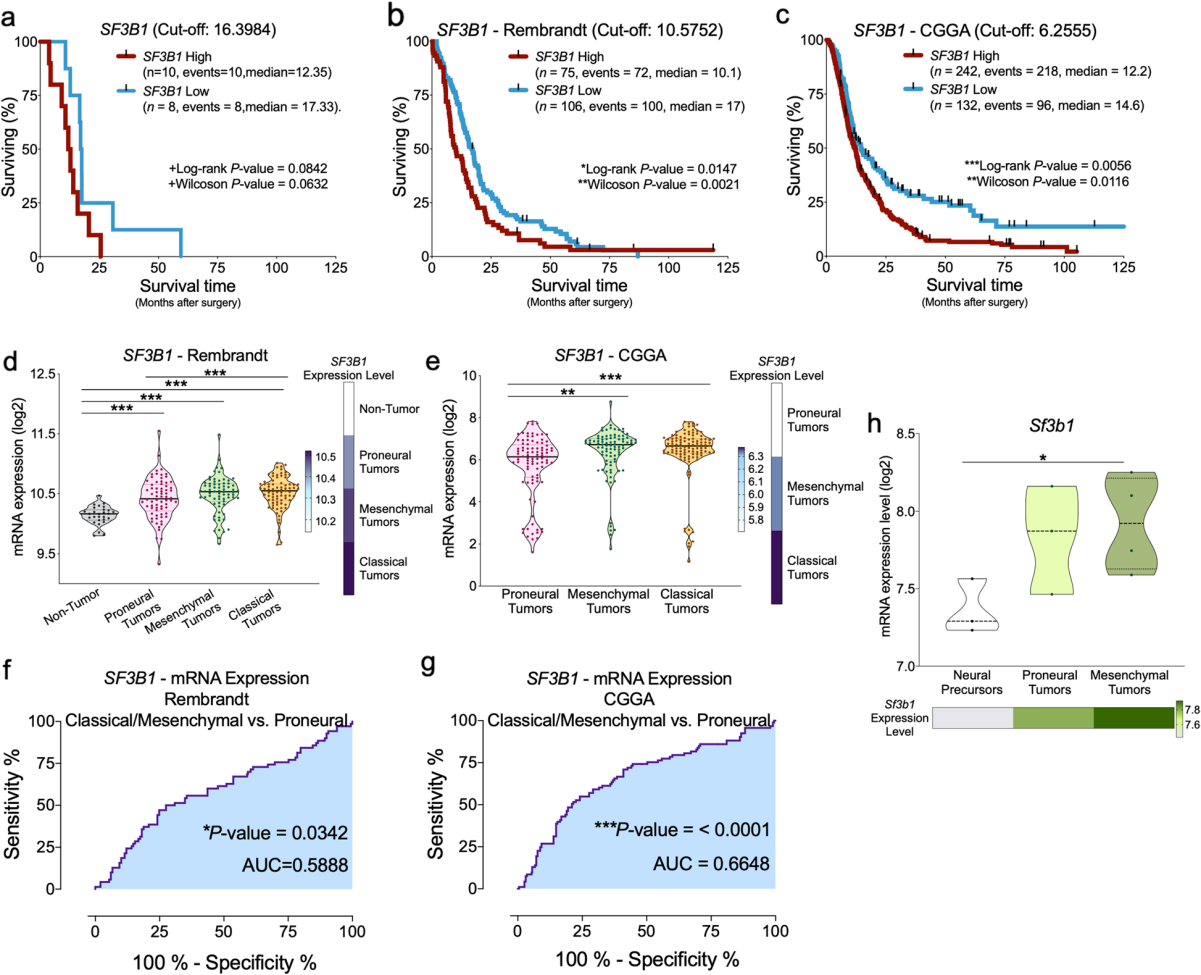
*SF3B1* overexpression is associated with poor survival. Kaplan-Meier survival curves for GBM patients with high and low expression levels of *SF3B1* in our cohort of patients (**a**), as well as in the Rembrandt (**b**) and CGGA (**c**) datasets. Comparison of expression levels of *SF3B1* and heatmaps generated using *SF3B1* levels between control samples and proneural, mesenchymal, and classical GBM subtypes from the Rembrandt (**d**) and CGGA (**e**) datasets. ROC-curve analyses of *SF3B1* comparing classical/mesenchymal GBM vs. proneural GBM samples in the Rembrandt (**f**) and CGGA (**g**) datasets. **h** *SF3B1* expression levels (*upper panel*) and heatmap (*lower panel)* discerning between neural precursor cells, proneural and mesenchymal-like tumors from EPed mouse models. Asterisks (*P < 0.05; **P < 0.01; ***P < 0.001) indicate statistically significant differences across different conditions. Plus symbol (+) indicates a tendency between conditions (+*P* > 0.05 < 0.1)

High *SF3B1* mRNA levels were strongly associated with a worse survival rate in GBM patients in our cohort (Fig. 4a), which was corroborated in two additional patient cohorts (Rembrandt- and CGGA-dataset; Fig. 4b-c). Remarkably, a higher *SF3B1* expression was found in human mesenchymal and classical GBM (both GBM -subtypes with poorer -survival) compared to control samples and/or to proneural GBM (GBM subtype with better- survival) in both Rembrandt (Fig. 4d) and CGGA (Fig. 4e) cohorts. Moreover, ROC-curve analyses reinforced the potential prognostic capacity of the *SF3B1* overexpression levels to significantly discriminate between classical/mesenchymal -GBM and proneural -GBM in both external patient cohorts [Rembrandt (Fig. 4f) and CGGA (Fig. 4g)]. Consistently, *Sf3b1* expression levels were also elevated in mesenchymal-like -GBM vs. control samples from neural precursors from the EPed mouse model (Fig. 4h), being its expression in mesenchymal-like GBM also higher than in proneural-like GBM but this latter difference did not reach statistical significance (Fig. 4h). Additionally, the Genomics of Drug Sensitivity in Cancer (GDSC) dataset was explored to analyze the potential implication of *SF3B1* in pharmacological resistance (Fig. S3a-b). These analyses revealed that the resistance to drugs targeting RTK signaling pathways, chromatin acetylation, DNA replication, cell cycle, and mTOR/PI3K signaling pathways was associated with *SF3B1* expression, which unveils the potential implication of the dysregulation of *SF3B1* in these oncogenic pathways to confer drug resistance in GBM (Fig. S3c; Tables S8–9).

### Pharmacological inhibition of SF3B1 with pladienolide B decreases functional and molecular aggressiveness parameters in vitro in GBM cells

*SF3B1* expression levels were significantly higher in U-87/U-118 MG cells compared with non-tumor brain tissues (Fig. S4a) and were slightly higher, but comparable, in U-87/U-118 MG cells and GBM samples, suggesting that both cell lines were appropriate GBM models to study SF3B1 functional role. Subsequently, dose-response experiments indicated that 100 nM of pladienolide B was the most effective concentration reducing proliferation rate in U-87/U-118 MG cells (Fig. S4b) and in primary-GBM cell cultures (Fig. S4c) after IC_50_ determination (Fig. S4d). Therefore, 100 nM-dose was selected for subsequent experiments.

**Fig. 5:**
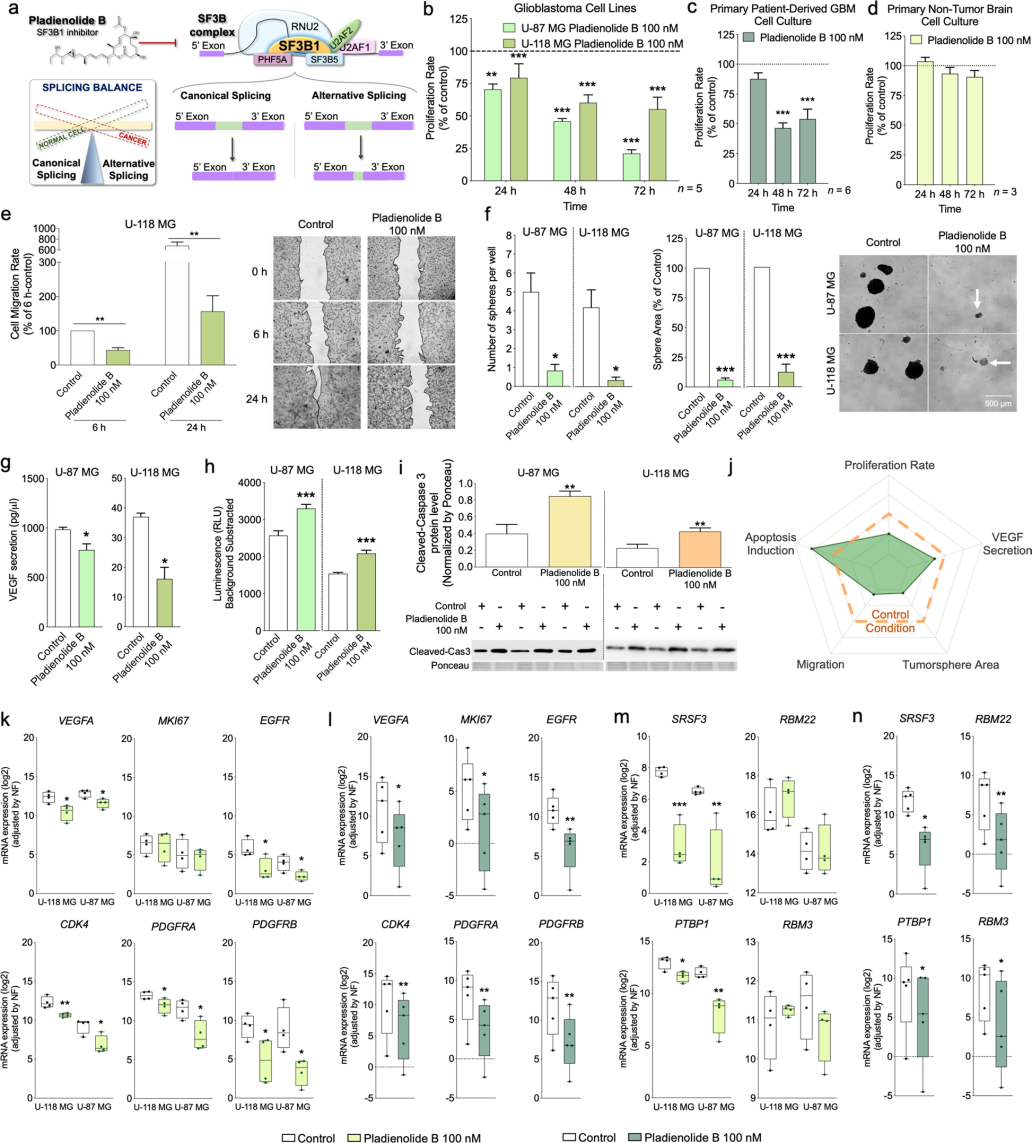
Pharmacological inhibition of SF3B1 with pladienolide B in vitro decreases critical functional parameters of aggressiveness and key tumor development/progression/aggressiveness markers in GBM cells compared to control conditions. **a** Schematic representation of the effect of pladienolide B inhibiting SF3B1. Proliferation rate in response to pladienolide B administration in GBM cell lines (U-87 MG and U-118 MG; *n* = 5) (**b**), in primary patient-derived GBM cells (*n* = 6) **(c)**, and in primary non-tumor brain cell cultures (*n* = 3) (**d**). **e** Migration rate after pladienolide B in U-118 MG cells (representative images of the migration capacity are also included; *n* = 5). **f** Tumorsphere formation assay showing sphere area and number of tumorspheres per well in response to pladienolide B administration in U-87 MG and U-118 MG cells (*n* = 3; representative images of tumorspheres formation are also included). **g** VEGF secretion in response to pladienolide B in U-87 MG and U-118 MG cells (*n* = 3). **h** Apoptosis rate after pladienolide B administration in U-87 MG and U-118 MG cells (*n* = 3). **i** Protein levels of cleaved-caspase 3 after 24 h of incubation with pladienolide B determined by western blot (*n* = 3). **j** Summary of the effect of pladienolide B treatment on the different functional parameters previously mentioned. Expression of different tumor progression markers after pladienolide B treatment in the two GBM cell lines (**k**) and in primary patient-derived GBM cells (**l**). Expression of critical oncogenic spliceosome components after pladienolide B treatment in the two GBM cell lines (**m**) and in primary patient-derived GBM cells (**n**). Asterisks (*P < 0.05; **P < 0.01; ***P < 0.001) indicate statistically significant differences across different conditions

Pharmacological SF3B1 blockade, which disrupts the spliceosome activity (Fig. 5a), significantly decreased proliferation rate in a time-dependent manner in both cell lines (Fig. 5b) and primary-GBM cell cultures (Fig. 5c), but not in primary non-tumor brain cell cultures (Fig. 5d), suggesting that pladienolide B effects are selectively exerted on GBM cells. In this sense, a positive correlation between *SF3B1* expression levels in the primary GBM cells cultures and the percentage of reduction of pladienolide B on proliferation rate was found (Fig. S4e-f). Therefore, we might speculate that pladienolide B is not effective in reducing proliferation rate in non-tumor cells due to the significantly lower expression levels of *SF3B1* compared with GBM cells; however, further studies would be necessary to unequivocally corroborate this idea. Furthermore, pladienolide B treatment also reduced the migration rate in U-118 MG cells at 6 h and 24 h (Fig. 5e). Furthermore, a tumorsphere formation assay (used to quantify the proliferation capacity of cancer stem-like progenitor cells) revealed that SF3B1 blockade drastically decreased the number and area of tumorspheres in both cell lines (Fig. 5f). Moreover, a decrease in VEGF secretion was observed after pladienolide B treatment in both cell lines (Fig. 5g). Capase3/7 luciferase-assay revealed that SF3B1 inhibition induced apoptosis in both cell lines (Fig. 5h), as also confirmed by an increase of cleaved-caspase 3 levels by western blot (Fig. 5i). All these results revealed that pladienolide B treatment affected different critical functional endpoints associated with the development, progression and aggressiveness of GBM cells (Fig. 5j).

Pladienolide B treatment also decreased the expression of key tumor progression markers and critical oncogenic spliceosome components [previously found to be correlated with *SF3B1* in GBM samples (Fig. 1l-m)] in both cell lines (Fig. 5k and m, respectively) and primary-GBM cell cultures (Fig. 5l and n, respectively).

### In vivo pharmacological inhibition of SF3B1 with pladienolide B impairs GBM progression and vascularization

**Fig. 6:**
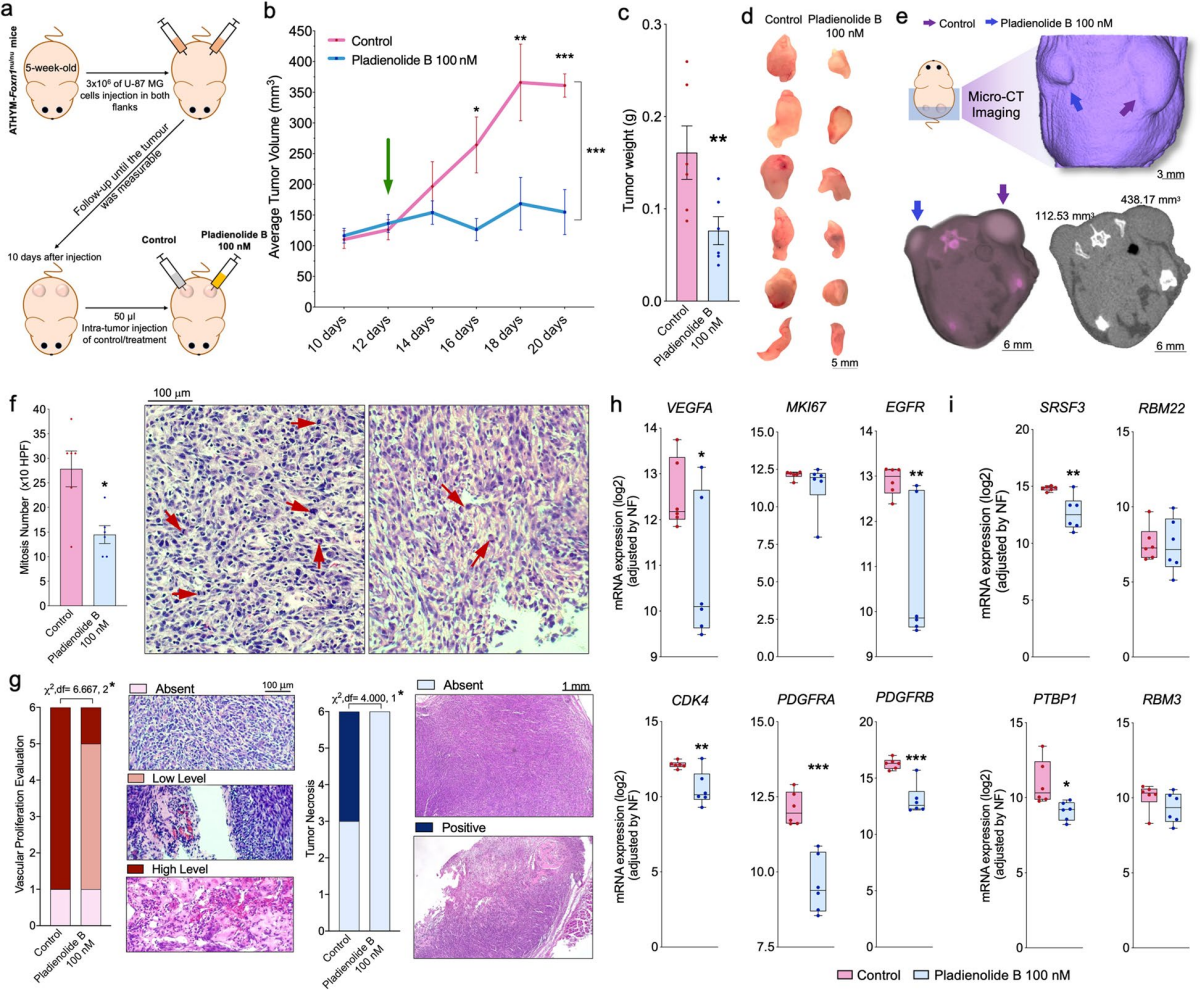
In vivo pharmacological inhibition of SF3B1 with pladienolide B impairs GBM progression and vascularization. **a** Generation of a preclinical-xenograft GBM model by inoculation of U-87 MG cells (*n* = 6). Average tumor volume (**b**) and weight (**c**) of intra-tumor pladienolide B injection vs. control-treated tumors. The green arrow in (**b**) indicates the moment of the corresponding treatment (intra-tumor injection with pladienolide B or control). **d** Images of each tumor at the moment of sacrifice are shown individually. **e** 2D- and 3D-micro-CT imaging of a representative preclinical-xenograft GBM -model. **f** Mitosis number (× 10 HPF; left panel) and representative images of H&E staining (right panel) comparing intratumor pladienolide B injection vs. control-treated tumor samples. **g** Vascular proliferation evaluation and representative images of H&E staining (left panel) as well as tumor necrosis evaluation and representative images of H&E staining (right panel) of intra-tumor pladienolide B injection vs. control-treated tumor samples. All these evaluations were determined by experienced pathologists. Expression of different tumor progression markers (**h**) and critical oncogenic spliceosome components (**i**) after pladienolide B treatment in the preclinical-xenograft GBM model. Asterisks (*P < 0.05; **P < 0.01; ***P < 0.001) indicate statistically significant differences across different conditions

Pladienolide B intra-tumor administration in vivo reduced tumor volume and weight compared with control-treated tumors in a preclinical-xenograft U-87 MG GBM model (Fig. 6a-d). Tumor volume clearly showed that GBM progression in vivo was completely stopped in pladienolide B treated tumors vs. control-treated tumors (that rapidly continued their progression; Fig. 6b). Moreover, 2D-micro-CT images together with 3D-rendering confirmed these in vivo differences (Fig. 6e). Furthermore, mitosis number was decreased in pladienolide B treated tumors vs. control-treated tumors (Fig. 6f). Additionally, pladienolide B treated tumors showed low levels of vascular proliferation (5/6 tumors) and absence of necrosis (all tumors) (Fig. 6g). As previously observed in vitro, pladienolide B administration in vivo significantly decreased various relevant tumor progression markers and critical oncogenic spliceosome components (*VEGFA/EGFR/CDK4/PDGFRA/PDGFRB and SRSF3/PTBP1*; Fig. 6h-i). Therefore, all these in vivo results (Fig. 6) support the antitumor effects of SF3B1 blockade previously observed in vitro (Fig. 5).

### Pre-treatment with pladienolide B in vitro affects the onset/formation of GBM tumors in vivo

**Fig. 7:**
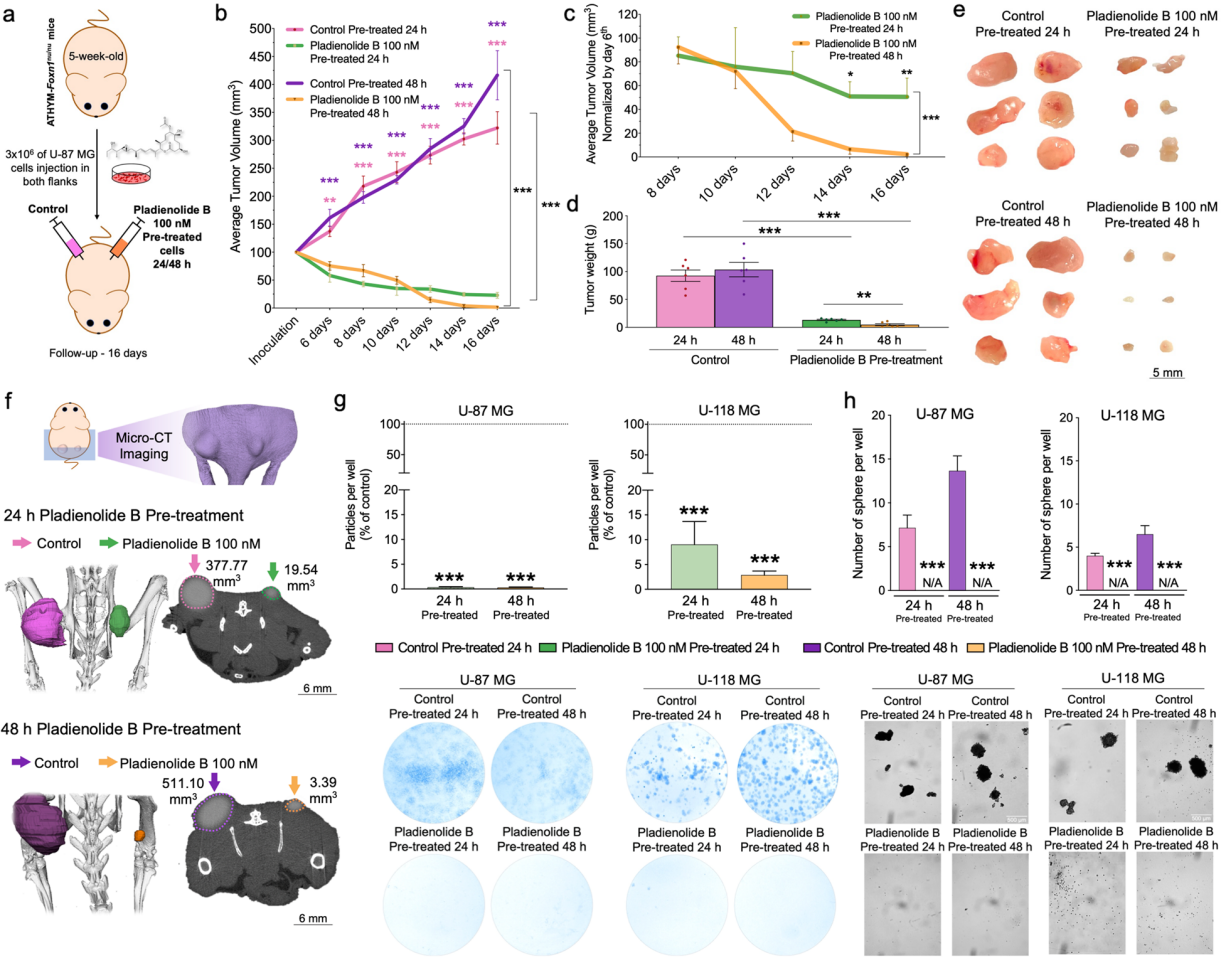
Pre-treatment with pladienolide B in vitro impairs the onset/formation of GBM tumors in vivo and reduces colony and tumorsphere formation in vitro. **a** Generation of a preclinical-xenograft GBM model by inoculating U-87 MG cells previously pre-treated with pladienolide B in vitro for 24 h (*n* = 6) and 48 h (*n* = 6) compared with control-treated cells. **b** Average tumor volume of control-treated vs. pladienolide B-treated cells [(**c**) comparison of tumor volume between xenograft GBM-model with pre-treated cells for 48 h vs. 24 h]. **d** Average weight of control-treated vs. pladienolide B-treated cells. **e** Images of each tumor at the moment of sacrifice are shown individually. **f** 2D- and 3D-micro-CT imaging of a representative preclinical-xenograft GBM model hosting cells pre-treated for 24 h and 48 h with pladienolide B. **g** Particles per well using the colony formation assay after pladienolide B treatment in vitro (24 h and 48 h) in U-87 MG and U-118 MG cells (*n* = 3; representative images of colonies are included). **h** Number of tumorspheres per well using the tumorsphere formation assay after pladienolide B treatment in vitro (24 h and 48 h) in U-87 MG and U-118 MG cells (*n* = 3; representative images of tumorspheres formation are also included). Asterisks (*P < 0.05; **P < 0.01; ***P < 0.001) indicate statistically significant differences across different conditions

Pre-treatment with pladienolide B in vitro for 24 h and 48 h in GBM cells was able to impair GBM onset/formation in an in vivo preclinical-xenograft U-87 MG GBM model (Fig. 7a-b). Specifically, average tumor volume and weight were impaired in vivo in the xenograft U-87 MG GBM model pre-treated with pladienolide B compared with control-treated tumors, being this effect more pronounced in pre-treated cells for 48 h vs. 24 h (Fig. 7b-e). 2D-micro-CT images together with 3D-rendering also confirmed these in vivo differences (Fig. 7f). Additionally, we demonstrated that GBM cells pre-treated with pladienolide B in vitro were not able to undergo colony formation and tumorsphere formation (Fig. 7g and h, respectively). Altogether, these data demonstrate that treatment with pladienolide B is able to impair the capacity of GBM cells to onset tumor formation in vitro and in vivo.

### *SF3B1* expression is strongly associated with relevant components of cancer-related pathways in GBM

**Fig. 8:**
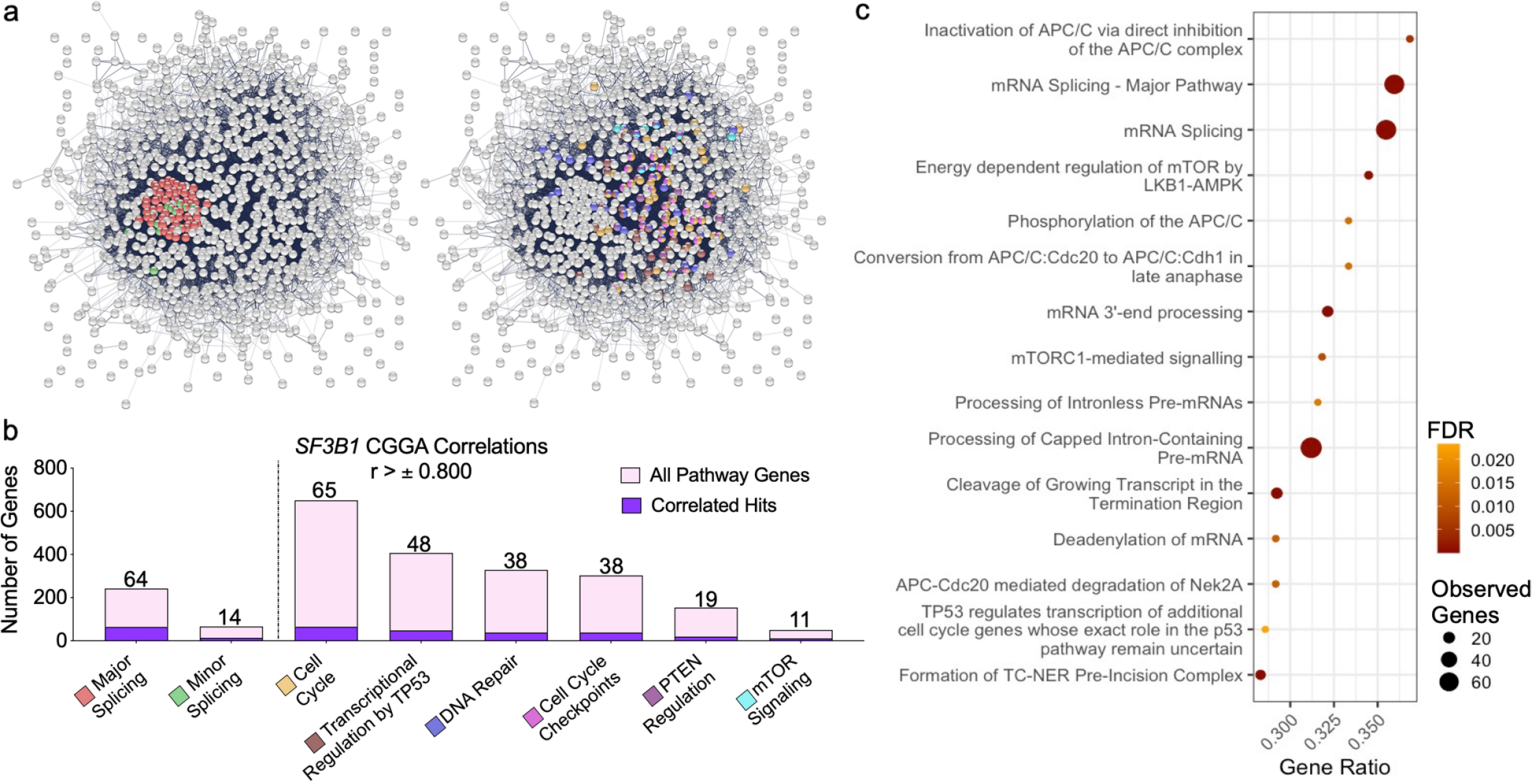
*SF3B1* is strongly related to certain cancer-related pathways. **a** Functional association network of the significantly correlated genes with *SF3B1* using the CGGA dataset. These significantly altered genes were analyzed using the STRING database, and (**b)** they are marked according to their KEGG pathways analysis. **c** Gene set analysis enrichment terms for the genesets within the Reactome pathways using *SF3B1* correlated genes (cut-off r > ± 0.800)

A specific analysis of highly correlated genes (r > ± 0.800; CGGA-dataset) using STRING-tool and KEGG-database revealed a clear link among several genes involved in the splicing -process and critical cancer-related pathways (i.e., cell cycle, transcriptional regulation, DNA repair, mTOR-signaling, etc.; Fig. 8a-b and Table S5), which further supported the relevance of SF3B1 in tumor -physiopathology. Indeed, a more detailed enrichment analysis using Reactome-database was used to identify the main pathways associated with *SF3B1* expression, which revealed that the splicing process and AKT-mTOR/ß-catenin signaling pathways were closely associated with *SF3B1* (Fig. 8c).

### Pharmacological blockade of SF3B1 reveals AKT-mTOR pathway as the major driver of pladienolide B antitumor actions in GBM cells

**Fig. 9:**
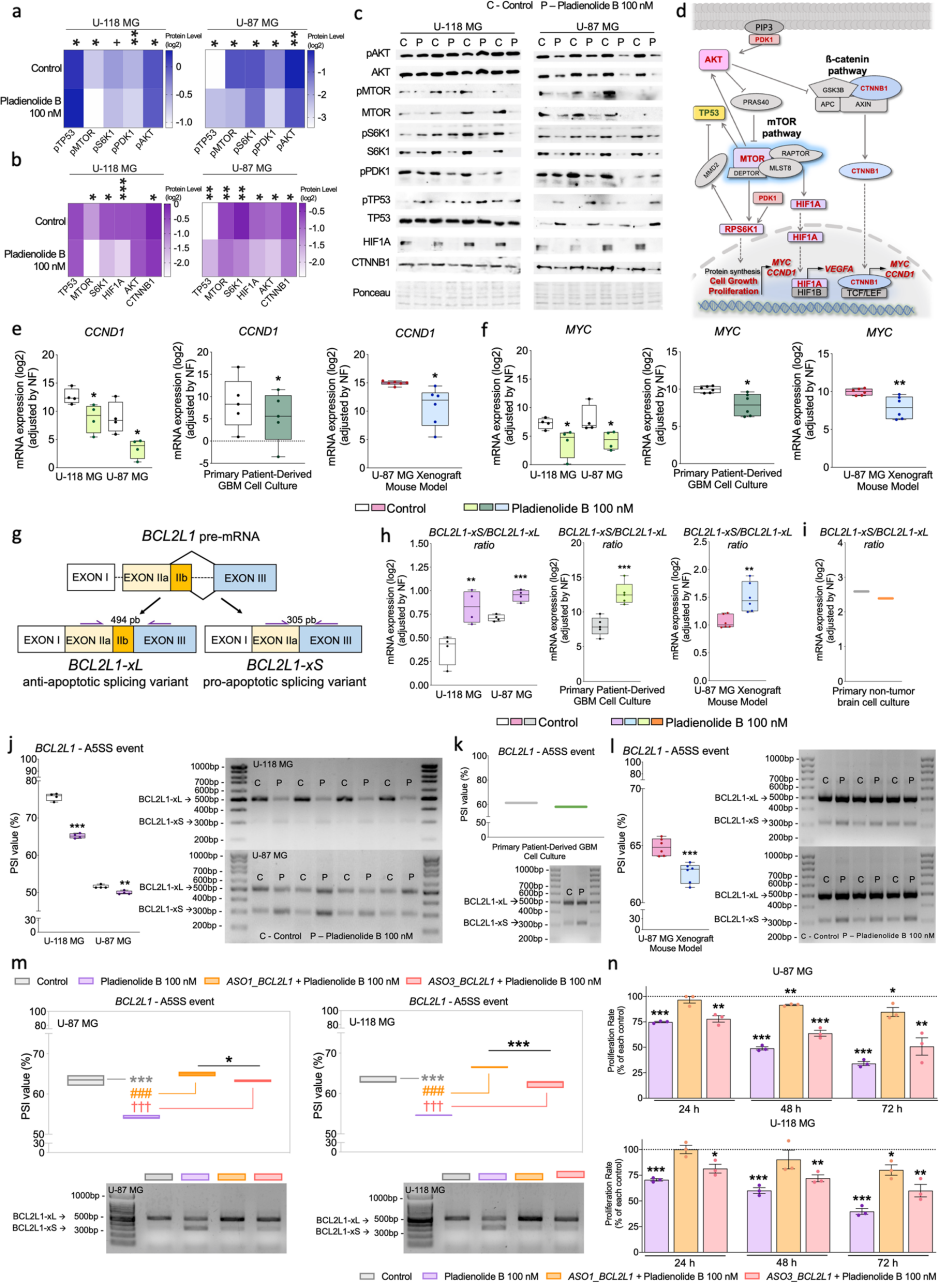
Pharmacological blockade of SF3B1 reveals AKT-mTOR and ß-catenin signaling pathways and BCL2L1 alternative splicing as major drivers of the pladienolide B antitumor effects in GBM. Heatmaps showing the western blot densitometric level (log2) of phosphorylated-proteins (**a**) and total-proteins levels (**b**) of several components of AKT/mTOR and ß-catenin pathways in GBM cells (U-87 MG and U-118 MG) after pladienolide B administration. **c** Images of western blot results showed in the previous heatmaps (**a**) and (**b**). **d** AKT-MTOR and ß-catenin pathways diagram showing the downregulated (in red) and upregulated (in the yellow box) components/processes after pladienolide B administration identified in this work. Expression levels of CCND1 (**e**) and MYC (**f**) as endpoints of AKT/mTOR and ß-catenin pathways in GBM cells (U-87 MG and U-118 MG), primary patient-derived GBM cells and in the preclinical-xenograft GBM model after pladienolide B administration. **g** *BCL2L1* splicing variants produced by an alternative 5′ spliced site (A5SS) splicing event and associated with apoptosis and cell death pathway. **h** *BCL2L1-xS/BCL2L1-xL* ratio determined by qPCR in GBM cell lines (U-87 MG and U-118 MG), in the preclinical-xenograft GBM model, and in primary patient-derived GBM cells in response to pladienolide B treatment. **i** *BCL2L1-xS/BCL2L1-xL* ratio determined by qPCR in primary non-tumor brain cell culture after pladienolide B administration. PSI of *BCL2L1* A5SS event in GBM cell lines (U-87 MG and U-118 MG) (**j**), in primary patient-derived GBM cells (**k**), and the preclinical-xenograft GBM model (**l**) in response to pladienolide B treatment. **m** Validation of designed antisense oligonucleotides (ASOs; *ASO1_BCL2L1* and *ASO3_BCL2L1*) by determination of PSI of *BCL2L1* A5SS event in GBM cell lines (U-87 MG and U-118 MG; *n* = 3). **n** Proliferation rate in GBM cells in response to control, pladienolide B, ASO1_BCL2L + pladienolide B, and *ASO3_BCL2L1* + pladienolide B cells (*n* = 3). The % has been calculated with the control, *ASO1_BCL2L1* and *ASO3_BCL2L1* transfected cells (without pladienolide B treatment) of each condition. Asterisks and symbols (*P < 0.05; **P < 0.01; ***/###/††† *P* < 0.001) indicate statistically significant differences across different conditions (i.e.; pladienolide B vs. control; *ASO1_BCL2L1+* pladienolide B vs. pladienolide B; *ASO2_BCL2L1+* pladienolide B vs. pladienolide B, respectively). Plus symbol (+) indicates a tendency between conditions (+P > 0.05 < 0.1)

Consistent with the previous data obtained in the present study with the enrichment analysis, pladienolide B regulated critical points of mTOR/TP53/AKT-pathway through the modulation of phosphorylated-protein levels (i.e., downregulation of pMTOR/pS6K1/pPDK1/pAKT and upregulation of pTP53; Fig. 9a; Fig. S5a) and total-protein levels (i.e., downregulation of AKT/MTOR/S6K1/CTNNB1/TP53/HIF1A; Fig. 9b; Fig. S5b) in U-87/U-118 MG cells (Fig. 9a-d). Furthermore, *CCND1* and *MYC* mRNA levels (two classical-endpoints of these signaling pathways associated with cell survival, growth, and proliferation) were measured in response to pladienolide B treatment in vitro and in vivo (Fig. 9d-f). Pladienolide B decreased *CCND1* and *MYC* expression levels in GBM cells (U-87/U-118 MG cells and primary-GBM cell -cultures) and the preclinical-xenograft GBM model (Fig. 9e-f).

Interestingly, pladienolide B treatment significantly decreased SF3B1 mRNA/protein levels in GBM cells (U-87/U-118 MG cells and primary-GBM cell cultures) and in the preclinical-xenograft GBM model (Fig. S5c-d). Moreover, given the reported implication of SRSF1 splicing factor with AKT, mTOR, and Wnt/ß-catenin pathways [22, 64, 74](Fig. 9d), we also interrogated the *SRSF1-*status [which was strongly correlated with *SF3B1, MTOR,* and *CCTNB1* expression (Fig. S5e)]. Specifically, *SRSF1* mRNA levels were decreased after pladienolide B administration in GBM cells in vitro (U-87/U-118 MG cells and primary-GBM cell -cultures) and the preclinical-xenograft GBM model (Fig. S5f).

### Changes in *BCL2L1* splicing variants (SVs) expression profile as a potential driver of SF3B1 blockade antitumor actions

We next explored whether SF3B1 pharmacological blockade, using pladienolide B altered the splicing process of some critical genes implicated in GBM progression which have been previously reported to be associated with cancer-related signaling -pathways (i.e., *KLF6/CRK/MST1R/CASP2/RAC1/MCL1/BIRC5/SPP1*/*BCL2L1*). Specifically, we performed a screening of the SVs of these genes in U-87/U-118 MG cells using end-point PCR-methodology (data not shown). Among them, only *BCL2L1* showed an alteration in the SV-profile (Fig. 9g-l). More specifically, *BCL2L1* has nine SVs, and two of them, *Bcl-xL* and *Bcl-xS*, are commonly reported to be associated with cancer [6, 10], being *Bcl-xL* an anti-apoptotic and oncogenic, while *Bcl-xS* acts as a pro-apoptotic tumor suppressor (Fig. 9g). We studied the balance of these two *BCL2L1*-variants, their regulation by PSI (Percent Spliced In index) analysis, and/or their presence by RT-qPCR. This latter analysis revealed that *Bcl-xS/Bcl-xL* ratio was elevated after SF3B1 inhibition with pladienolide B in GBM in vivo (i.e., preclinical-xenograft GBM model) and GBM cells in vitro (U-87/U-118 MG cells and primary-GBM cell cultures) (Fig. 9h), but not in an available primary non-tumor cell cultures (Fig. 9i). Particularly, anti-apoptotic *Bcl-xL* was significantly downregulated while pro-apoptotic *Bcl-xS* was upregulated after pladienolide B treatment in the preclinical-xenograft GBM model (Fig. S5g), GBM cells [U-87/U-118 MG cells (Fig. S5h) and primary-GBM cell cultures (Fig. S5i)], but not in primary non-tumor cell cultures (Fig. S5j). Likewise, PSI determination confirmed previous results since pladienolide B treatment reduced *BCL2L1* alternative 5′ end splice site (A5SS) splicing event in U-87/U-118 MG cells (Fig. 9j), primary-GBM cell -cultures (Fig. 9k), and in the preclinical-xenograft GBM model (Fig. 9l). Additionally, the ability of SF3B1 to influence *BCL2L1*-splicing was further substantiated by the fact that PSI values were directly correlated with *SF3B1 *expression in U-87/U-118 MG cells (Fig. S5k).

In order to corroborate that the alternative splicing dysregulation of *BCL2L1* is a potential driver of pladienolide B-mediated antitumor effects, we designed, validated, and used different ASOs that might be able to revert the *Bcl-xS/Bcl-xL* splicing process observed in response to pladienolide B. First, we performed an initial screening using U-87 MG cells to optimize the ASOs transfection and to identify which of the 5 designed ASOs were efficient in inhibiting the pro-apoptotic *Bcl-xS* variant and in promoting the anti-apoptotic *Bcl-xL* variant in response to pladienolide B treatment. Among the 5 designed ASOs, only two (*ASO1_BCL2L1* and *ASO3_BCL2L1*) were able to efficiently revert the splicing process of *BCL2L1* after pladienolide B treatment (Fig. 9m). Specifically, as previously observed, pladienolide B treatment significantly downregulated the anti-apoptotic *Bcl-xL* variant while upregulated the pro-apoptotic *Bcl-xS* variant in intact (non-transfected) GBM cells (Fig. 9m). In contrast, the pro-apoptotic *Bcl-xS* variant was significantly inhibited while the anti-apoptotic *Bcl-xL* variant was upregulated in ASO-transfected GBM cells (*ASO1_BCL2L1* and *ASO3_BCL2L1*) treated with pladienolide B (Fig. 9m). Similar results were then confirmed in both cell lines (U-87 MG and U-118 MG; *n* = 3) transfected with *ASO1_BCL2L1* and *ASO3_BCL2L1* and treated with pladienolide B (Fig. 9m). Therefore, these results revealed that the transfection with both ASOs in GBM cells treated with pladienolide B was able to revert the splicing process of *BCL2L1* to the same level as control-treated cells. Then, we tested if this ASO-mediated inhibition of the *BCL2L1* pro-apoptotic variant following pladienolide B treatment was able to reduce the antitumor effect of pladienolide B on GBM growth using a proliferation assay. Specifically, the results uncovered that the proliferation rate of ASO-transfected GBM cells (*ASO1_BCL2L1* and *ASO3_BCL2L1*) in response to pladienolide B treatment was significantly blunted compared with non-transfected cells treated with pladienolide B (Fig. 9n). Particularly, *ASO1_BCL2L1* seems to be more effective than *ASO3_BCL2L1* in impairing the antitumor effect of pladienolide B which might be explained by the significant low PSI observed in the cells transfected by *ASO3_BCL2L1* vs. *ASO1_BCL2L1* (Fig. 9m). Altogether, these data suggest that alternative splicing dysregulation of *BCL2L1* seems to be a potential driver of pladienolide B-mediated antitumor effects.

## Discussion

Targeting the spliceosome machinery could become an innovative and successful therapeutic approach to treat incurable cancers like GBM. Indeed, the transcriptomic landscape of cancer cells makes them particularly vulnerable to pharmacological inhibition of splicing, which might have important therapeutic relevance in the near future as suggested by multiple ongoing clinical trials aimed to answer this question [7]. Specifically, various drugs have been designed to target SF3B1 (a central/essential core-component of the spliceosome) [16, 35, 48], making this spliceosome element the best candidate to study its translational oncogenic implication and therapeutic capacity in cancers wherein there are no successful treatments or cure. However, the data published so far focused on the potential oncogenic role and therapeutic effectiveness of the modulation of critical spliceosome components through pharmacological approaches is quite limited, fragmentary, and unclear [7, 48, 52]. To the best of our knowledge, the oncogenic implication and therapeutic capacity of SF3B1, its somatic mutations, and expression profile have not been characterized in GBM, neither its association with molecular features nor clinical parameters.

Herein, we demonstrated that SF3B1 dysregulation clearly affects several cancer hallmarks including apoptosis/proliferation/migration/angiogenesis/splicing pattern and signaling among others, and, of particular clinical relevance, that it could be associated with the development of drug resistance. Since splicing perturbations are common in cancer, including brain tumors [23], and are associated with mutations and/or altered expression of splicing machinery [23, 34, 70], we determined the *SF3B1*mut-frequency and whether these mutations were associated with glioma progression. Interestingly, *SF3B1*mut-frequency was low in glioma patients (~ 1%) compared to other cancer pathologies [wherein *SF3B1*mut range from 5% in breast cancer to 81% in myelodysplastic syndromes [7, 36, 69]]. Moreover, no difference was observed in mean OS of glioma patients with *SF3B1*mut compared to *SF3B1*wt, an observation that is not similar to previous data in chronic lymphocytic leukemia indicating that *SF3B1*mut are associated with rapid disease progression and unfavorable OS [69]. The low *SF3B1*mut-frequency found in glioma patients might suggest that the potential SF3B1role in glioma pathogenesis could be exerted through altered expression levels rather than somatic mutations. In fact, we demonstrate for the first time a drastic SF3B1 overexpression (at mRNA/protein levels) in different cohorts of GBMs vs. non-tumor tissues, which was also confirmed in EPed-glioma mouse models vs. control samples. Moreover, bioinformatic analyses revealed a potential diagnostic capacity of SF3B1 levels to discriminate between GBM/gliomas vs. control tissues from humans and mice, suggesting that GBM/glioma curse with a global dysregulation of SF3B1 in different species. Furthermore, our data revealed a potential utility of SF3B1 as aggressiveness biomarker in GBM which is supported by the direct and strong association found between SF3B1 expression levels and relevant development/progression tumor -markers (e.g., MKI67*/PDGFRA*) [59] and different oncogenic spliceosome components, including *SRSF3* (the most critical splicing machinery component in GBM recently identified by our group) [23], in human GBM and tumor -samples from EPed-glioma mouse models.

Most importantly, this study revealed that high *SF3B1* expression is directly associated with a worse OS rate in GBM patients, certainly, the main clinical problem in this pathology. This finding was corroborated in two external patient cohorts with GBM (Rembrandt/CGGA-dataset) and further supported by similar observations found in other tumor pathologies [3, 4, 32, 38, 49, 63]. Similarly, a higher *SF3B1* expression was observed in human and mouse classical and mesenchymal GBM (subtypes with poorer survival rate) compared to proneural GBM (subtype with better survival rate) or non-tumor samples, which reinforced the prognostic value and potential oncogenic role of *SF3B1* [68]. To the best of our knowledge, this is the first report identifying the diagnostic and prognostic capacity of *SF3B1* in human GBM, and in glioma mouse models with different prognoses, wherein these observations suggest a causal link between *SF3B1 *dysregulation and GBM aggressiveness. Notably, we also characterized *SF3B1 *expression at the single-cell level demonstrating *SF3B1* was homogeneously expressed across all GBM cell populations/states, being higher in cells expressing a proliferative neural progenitors-like transcriptional program. These data are therapeutically important and have a potential translational/oncogenic implication since the current therapeutic strategies for GBM are not efficient at reducing tumor volume/growth or augmenting survival rate, which is likely due, in part, to the resistance acquired by tumors, particularly by neural progenitors-cells, to different current drugs [51]. Therefore, our data showing that *SF3B1*, a druggable spliceosome component, is homogeneously overexpressed in all GBM cell populations/states offer a novel opportunity and therapeutic approach to treat GBM. Remarkably, GDSC-dataset analysis also unveiled a potential implication of *SF3B1 *dysregulation in different oncogenic pathways (e.g., mTOR-PI3K/cell cycle/DNA replication, etc.) to confer drug resistance in GBM, which further encourages the use of an SF3B1 specific-inhibitor in GBM.

Indeed, we demonstrate strong in vitro/in vivo antitumor actions of pladienolide B in GBM cells. Notably, SF3B1 blockade induced marked reductions in aggressiveness features of different GBM cell models [cell -lines and primary-GBM cell -cultures, i.e., inhibition of proliferation/migration/VEGF secretion, and increase of apoptosis]. Most notably, SF3B1 blockade strikingly decreased also GBM-stem/progenitor cells in terms of tumorspheres number and area, both relevant functional results that may help to explore the GBM onset and how to overcome the well-known GBM -resistance to different/current drugs [21, 51]. It should be emphasized that our data also suggest that pladienolide B effects selectively impact on GBM cells and not non-tumor brain cells, which is clinically relevant and agrees with previous data in other cancer-types, where splicing inhibitors exert stronger, more selective actions on cancer cells than on non-transformed cells [14]. Moreover, we demonstrate that SF3B1 is also an effective target in GBM in vivo since pladienolide B treatment effectively blocks GBM progression of already established GBM tumors and the GBM onset/formation in preclinical GBM mouse models. Indeed, pladienolide B clearly blunted tumor volume compared to control tumors (which drastically continued their progression), and markedly decreased tumor-weight/mitosis-number of GBM cells and vascularization and necrosis in vivo. Furthermore, pharmacological SF3B1 blockade also decreased the expression of key tumor progression markers and critical oncogenic spliceosome components in GBM cells in vitro and in vivo. Remarkably, pre-treatment with pladienolide B in vitro (24/48 h) was also capable to impair the in vivo onset/formation of GBM possibly through disruption of GBM stem-cell survival. Thus, all these robust in vitro/in vivo results, together with the extended OS observed in different human cohorts, unveiled an important pathophysiological role of SF3B1 in GBM. Although some aspects should be considered when using pladienolide B (e.g., specific concentration used, possible side effects in patients, etc), our data suggest that SF3B1 blockade could be a novel therapeutic avenue with relevant pathophysiological/clinical-potential to combat this devastating disease.

We also interrogated the signaling mechanisms underlying the antitumor actions of SF3B1 blockadge in GBM in vitro and in vivo. Our data revealed, for the first time in GBM, a striking alteration in relevant routes closely associated with GBM progression and initiation, especially the AKT-mTOR and ß-catenin signaling pathways [12, 37, 46, 56], in response to SF3B1 blockade. In support of the link between SF3B1 activity and AKT pathway, it has been recently reported that *SF3B1*^*K700E*^ mutation can modulate the expression of key components of the AKT pathway with resulting increases in the migration/invasion of breast cancer cells [39]. Specifically, we observed an overall downregulation in several critical points belonging to these pathways [i.e., total-protein -levels of AKT/MTOR/S6K1/CTNNB1/TP53/HIF1A; phosphorylated-protein levels of AKT/MTOR/S6K1/PDK1 and expression levels of *CCND1* and *MYC*) and an upregulation of phosphorylated-TP53 levels, in different GBM models (in vitro and/or in vivo) in response to SF3B1 blockade. Moreover, our data indicate that pladienolide B inhibitory actions observed in AKT-mTOR/ß-catenin signaling pathways may likely be exerted through a significant down-regulation in *SRSF1*-levels, a relevant pro-oncogene overexpressed in GBM [1, 13, 23, 71, 74] which acts activating both signaling pathways simultaneously [22, 64]. This idea is further supported by the fact that SRSF1 and SF3B1 are functionally connected since SRSF1 directly interacts with the U2-snRNP complex where SF3B1 takes part [15], and by our data indicating that *SRSF1 *expression is strongly correlated with *SF3B1*, *MTOR,* and *CCTNB1* expression in GBM. Therefore, these data provide original, compelling evidence that SF3B1 is functionally linked, likely via SRSF1 modulation, to these well-known relevant pro-oncogenic pathways (AKT-mTOR/ß-catenin) in GBM, which further supports the pathophysiological relevance of SF3B1 and the antitumor actions of SF3B1-blockade in GBM. Interestingly, SF3B1 blockade suppressed *SF3B1* -expression suggesting positive feedback that could enhance its antitumor effects.

SF3B1 blockade also exerted important molecular actions involving the splicing modulation of two clinically relevant SVs of *BCL2L1* (*Bcl-xL* and *Bcl-xS*) associated with cancer -development and known to play an oncogenic role and a tumor suppressor actions, respectively [6, 10]. Specifically, SF3B1-blockade downregulated anti-apoptotic *Bcl-xL*, while upregulated the pro-apoptotic *Bcl-xS*, in GBM, both in vitro and in vivo, but not in non-tumor brain cell cultures. This idea was further corroborated by PSI analysis demonstrating a pladienolide B-induced reduction of *BCL2L1* A5SS splicing -event in GBM in vitro and in vivo. In line with these data, previous reports found that several apoptosis-regulatory genes, including *BCL2*-related genes, generate alternatively SVs with opposite activities, which is a biological program often employed by cancer -cells to escape from intrinsically programmed cell death and radiotherapy/chemotherapy-induced cytotoxicity [72]. In fact, our observations in *Bcl-xL/xS,* together with the data demonstrating the implication of *SF3B1 *dysregulation in different oncogenic -pathways that confers drug resistance in GBM (e.g., mTOR-PI3K/cell cycle/DNA -replication, etc.), might be clinically relevant because it has been demonstrated that *Bcl-xL* is transcriptionally upregulated and associated with poor prognosis and chemoresistance in many cancers [6, 10]. In this sense, it should be indicated that the use of two different ASOs that inhibited the pro-apoptotic *Bcl-xS* variant and promoted the anti-apoptotic *Bcl-xL* variant in response to pladienolide B treatment was able to significantly reduce the antitumor effect of pladienolide B on GBM cells. All these data demonstrate that changes in the splicing of *BCL2L1* seem to be one of the main molecular mechanisms underlying the link between SF3B1 blockade and the significant decrease in GBM onset, GBM progression, and aggressiveness features observed in response to pladienolide B treatment.

## Conclusions

Taken our evidences together, our results unveiled new conceptual and functional avenues in GBM, with potential clinical implications, by demonstrating that SF3B1 is an attractive therapeutic target in GBM since its inhibition impaired key pathophysiological processes in GBM -biology (i.e., proliferation/migration/tumorspheres formation/apoptosis, etc.) likely by modulating different oncogenic signaling pathways (AKT-mTOR/ß-catenin) associated with GBM survival/initiation/progression, and an imbalance of *BCL2L1* splicing. Moreover, we found that *SF3B1* overexpression in GBM is associated with key molecular and clinical features including overall survival, poor prognosis, and drug resistance. Therefore, these results point out SF3B1 as a potential diagnostic/prognostic biomarker and a promising pharmacological target to treat patients with GBM, offering a clinically relevant opportunity that should be tested for use in humans.

## Supplementary Information

**Additional file 1: Figure S1.** Single-cell bioinformatic analyses to characterize intra-tumor cell populations **(a)** Quality control (QC) metrics plots showing the number of features, counts, and percentage of mitochondrial features in the single-cell RNAseq dataset and, **(b)** their features-counts/mithocondrial percentage relationship. **(c)** JackStraw Plot comparing the distribution of *P*-values for each principal component (PC) with a uniform distribution (dashed line). **(d)** Ranking of PC based on the percentage of variance. **(e)** Top3 markers were used to characterize each cluster of cells identified in the single-cell RNAseq dataset. **(f)** Non-hierarchical heatmap generated using the expression levels of *SF3B1* across the different TME cells and tumor-like cells subtypes. **(g)** Validation of Ponceau staining as a suitable internal control in U-87/U-118 MG cell lines in the two experimental conditions (control vs. pladienolide B) used in this study. Left images: Comparison of the signal obtained with Ponceau staining and western blot with anti-Beta-Actin (ACTB) and anti-Beta-tubulin (TUBB) in GBM cell models (U-87/U-118 MG) treated with pladienolide B and control. Right-top panel: Results showing that Ponceau, ACTB, and TUBB are not altered across experimental conditions (control vs. pladienolide B). Right-bottom panel: Results showing that the signal of TUBB or ACTB normalized by Ponceau staining is not altered across experimental conditions (control vs. pladienolide B). ns indicates non-statistically significant differences across different conditions. **Figure S2.** Somatic mutation rate of *SF3B1* as well as of commonly mutated genes in glioma samples (*IDH1, TP53, ATRX, PTEN, IDH2*) obtained from the TCGA-dataset (*n* = 476) **(a)**, and MSKCC-dataset (*n* = 841) **(b)**. Mutation count, glioma subtype, and grade are also shown. Kaplan-Meier survival curves for glioma patients with mutated and wildtype *SF3B1* obtained from the CGGA-dataset **(c)**, TCGA-dataset **(d)**, and MSKCC-dataset **(e)**. mRNA levels from *SF3B1* in control and GBM samples in our cohort of patients **(f)** and the external Rembrandt cohort **(g)**. Asterisks (*P < 0.05; ***P < 0.001) indicate statistically significant differences across different conditions. **Figure S3.** Analysis of the potential implication of *SF3B1* in pharmacological resistance using the Genomics of Drug Sensitivity using a Cancer dataset. **(a)** Heatmap generated by the number of drugs [Hits, Zscore > + 2 (cell line resistance)] and their association with *SF3B1* expression of different GBM cell lines. **(b)** Heatmap generated by the number of drugs [Hits, Zscore > − 2 (cell line sensitivity)] and their association with *SF3B1* expression of different GBM cell lines. **(c)** Pathway annotation of different drugs hit in **(a)** and **(b)**. **Figure S4.** Determination and accuracy of in vitro models and pladienolide B dose. **(a)** Comparison of the mRNA levels of *SF3B1* between non-tumor brain tissues, GBM tissues, and GBM cell lines U-87 MG and U-118 MG. **(b)** Dose-response of pladienolide B in GBM cell lines (U-87 MG and U-118 MG; *n* = 4) and **(c)** in primary patient-derived GBM cells. **(d)** IC_50_ of pladienolide B in vitro in the two GBM cell lines and primary patient-derived GBM cells. **(e)** Stratification of the primary GBM cell cultures used in the present study (*n* = 6) based on the percentage of reduction in the proliferation rate in response to pladienolide B (from lower to higher reduction; top-heatmap) and on the SF3B1 expression levels in the same primary GBM cell cultures (bottom-heatmap). **(f)** Correlation between SF3B1 expression levels and the effect of pladienolide B (48 h of incubation) in terms of proliferation rate in the primary GBM cell cultures used in the present study. Asterisks (*P < 0.05; **P < 0.01; ***P < 0.001) indicate statistically significant differences across different conditions. **Figure S5.** Unveiling the potential drivers of pharmacological blockade of SF3B1. **(a)** Heatmap showing the phosphorylated-protein level and **(b)** total-protein levels of several components of AKT/mTOR and ß-catenin pathways in GBM cells (U-87 MG and U-118 MG) after pladienolide B administration. **(c)** *SF3B1* mRNA levels after pladienolide B administration in GBM cell lines U-87 MG and U-118 MG, in primary patient-derived GBM cells and the preclinical-xenograft GBM model. **(d)** Protein levels of SF3B1 after pladienolide B administration in GBM cell lines U-87 MG and U-118 MG. **(e)** Correlation of *SRSF1* with *SF3B1*, *MTOR,* and *CCTNB1* using CGGA-dataset. **(f)** *SRSF1* mRNA levels after pladienolide B administration in GBM cell lines U-87 MG and U-118 MG, primary patient-derived GBM cells, and in the preclinical-xenograft GBM model. **(g)** *BCL2L1-xS* and *BCL2L1-xL* determined by qPCR in response to pladienolide B treatment in the preclinical-xenograft GBM model, **(h)** in GBM cell lines (U-87 MG and U-118 MG), and **(i)** in primary patient-derived GBM cells. **(j)** *BCL2L1-xS* and *BCL2L1-xL* determined by qPCR after pladienolide B administration in a primary non-tumor brain cell culture. **(k)** Correlation of *SF3B1* expression with *BCL2L1* A5SS PSI in GBM cell lines (U-87 MG and U-118 MG) after pladienolide B administration. Asterisks (*P < 0.05; **P < 0.01; ***P < 0.001) indicate statistically significant differences across different conditions. **Table S1.** Clinical characterization of patients with gliomas obtained from the CGGA-dataset, TCGA-dataset and MSKCC-dataset (WEseq) to detect SF3B1 somatic mutations. **Table S2.** Clinical characterization of patients with GBM from three different cohorts (our cohort, Rembrandt and CGGA datasets). **Table S3.** Top 10 cellular markers used to identify different clusters identified in single-cell RNA seq dataset. **Table S4.** Clinical characterization of patients with GBM obtained from the CPTAC dataset. **Table S5.** Results of enrichment analysis from CGGA-dataset using *SF3B1* high correlated genes (r > ± 0.800). Enrichment analysis was performed based on KEGG Pathways Analysis. **Table S6.** Specific primers for human transcripts used in this study were specifically designed and used in qPCR-based microfluidic assays. The official name of the genes, NCBI accession number of the transcripts, primers sequences, and product sizes of the amplification products are included. **Table S7.** Antisense Oligonucleotide (ASO) sequences for human *BCL2L1* were used in this study to specifically promote the splicing of *Bcl-xL* variant. The 3′-5′ ASO sequence [2′ O-methoxy-ethyl (MOE) residues are shown in bold)], 5′-3′ *BCL2L1* Targeted Region, and oligo sizes are included. **Table S8.** Drug resistance hits together with its corresponding annotated targeted pathway in *SF3B1* high-expression cell lines using the Genomics of Drug Sensitivity in Cancer dataset. **Table S9.** Drug sensitive hits together with its corresponding annotated targeted pathway in *SF3B1* low-expression cell lines using the Genomics of Drug Sensitivity in Cancer dataset.

## Data Availability

The datasets used and/or analyzed during the current study are available from public repositories indicated in the Methods section and/or from the corresponding author on reasonable request.

## Abbreviations

ANOVA: ANalysis Of VAriance
ASO: AntiSsense Oligonucleotide
A5SS: Alternative 5′ end Splice Site
ATCC: American Type Culture Collection
AUC: Area Under the Curve
CCLE: Cancer Cell Line Encyclopedia
D-MEM: Dulbecco´s Modified Eagle Medium
TCGA: The Cancer Genome Atlas
CGGA: Chinese Glioma Genome Atlas
CPTAC: Clinical Proteomic Tumor Analysis Consortium
EGF: Epidermal Growth Factor
EPed mouse models: Electro Porated mouse models
FBS: Fetal Bovine Serum
FFPE: Formalin-Fixed Paraffin-Embedded
GBM: Glioblastoma
GDSC: Genomics of Drug Sensitivity in Cancer
H&E-stain: Hematoxylin and Eosin Stain
IDH1: Isocitrate DeHydrogenase 1
IC_50_: Inhibition Concentration 50%
IHC assay: Immuno Histo Chemical assay
ISS: Intron Splicing Silencer
Micro-CT imaging: Micro-Computed Tomography imaging
mRNA: messenger RNA
NPC-like cells: Neural Precursor Cell-like cells
OPC-like cells: Oligodendrocyte Progenitor Cell-like cells
OS: Overall Survival
PBS: Phosphate-Buffered Saline
PCA: Principal Component Analysis
PCR: Polymerase Chain Reaction
PSI: Percent Spliced In index
ROC-curve: Receiver-Operating-Characteristic-curve
RT-qPCR: Reverse Transcription-quantitative PCR
RTK signaling pathways: Receptor Tyrosine Kinase signaling pathways
SDS-DTT: Sodium Dodecyl Sulfate DiThioThreitol
SF3B1: Splicing-Factor-3b-Subunit-1
SF3B1mut: SF3B1 mutated
SF3B1wt: SF3B1 wildtype
S-MEM: Spinner Minimun Essential Medium
SV: Splicing Variant
TME: Tumor MicroEnvironment
UMAP: Uniform Manifold Approximation and Projection
UCSC: University of California Santa Cruz
VEGF: Vascular Endothelial Growth Factor
WEseq: Whole Exome sequencing
2′ MOE: 2′ O-MethOxy-Ethyl
