# Supplementary material for: SF3B1 inhibition disrupts malignancy and prolongs survival in glioblastoma patients through BCL2L1 splicing and mTOR/ß-catenin pathways imbalances
Source: J Exp Clin Cancer Res. 2022 Jan 27;41:39. doi: 10.1186/s13046-022-02241-4 (PMC8793262; doi:10.1186/s13046-022-02241-4)
Supplement: Supplementary file 1 — Additional file 1: Figure S1. Single-cell bioinformatic analyses to characterize intra-tumor cell populations (a) Quality control (QC) metrics plots showing the number of features, counts, and percentage of mitochondrial features in the single-cell RNAseq dataset and, (b) their features-counts/mithocondrial percentage relationship. (c) JackStraw Plot comparing the distribution of P-values for each principal component (PC) with a uniform distribution (dashed line). (d) Ranking of PC based on the percentage of variance. (e) Top3 markers were used to characterize each cluster of cells identified in the single-cell RNAseq dataset. (f) Non-hierarchical heatmap generated using the expression levels of SF3B1 across the different TME cells and tumor-like cells subtypes. (g) Validation of Ponceau staining as a suitable internal control in U-87/U-118 MG cell lines in the two experimental conditions (control vs. pladienolide B) used in this study. Left images: Comparison of the signal obtained with Ponceau staining and western blot with anti-Beta-Actin (ACTB) and anti-Beta-tubulin (TUBB) in GBM cell models (U-87/U-118 MG) treated with pladienolide B and control. Right-top panel: Results showing that Ponceau, ACTB, and TUBB are not altered across experimental conditions (control vs. pladienolide B). Right-bottom panel: Results showing that the signal of TUBB or ACTB normalized by Ponceau staining is not altered across experimental conditions (control vs. pladienolide B). ns indicates non-statistically significant differences across different conditions. Figure S2. Somatic mutation rate of SF3B1 as well as of commonly mutated genes in glioma samples (IDH1, TP53, ATRX, PTEN, IDH2) obtained from the TCGA-dataset (n = 476) (a), and MSKCC-dataset (n = 841) (b). Mutation count, glioma subtype, and grade are also shown. Kaplan-Meier survival curves for glioma patients with mutated and wildtype SF3B1 obtained from the CGGA-dataset (c), TCGA-dataset (d), and MSKCC-dataset (e). mRNA leve [file 13046_2022_2241_MOESM1_ESM.pdf]

Figure S1

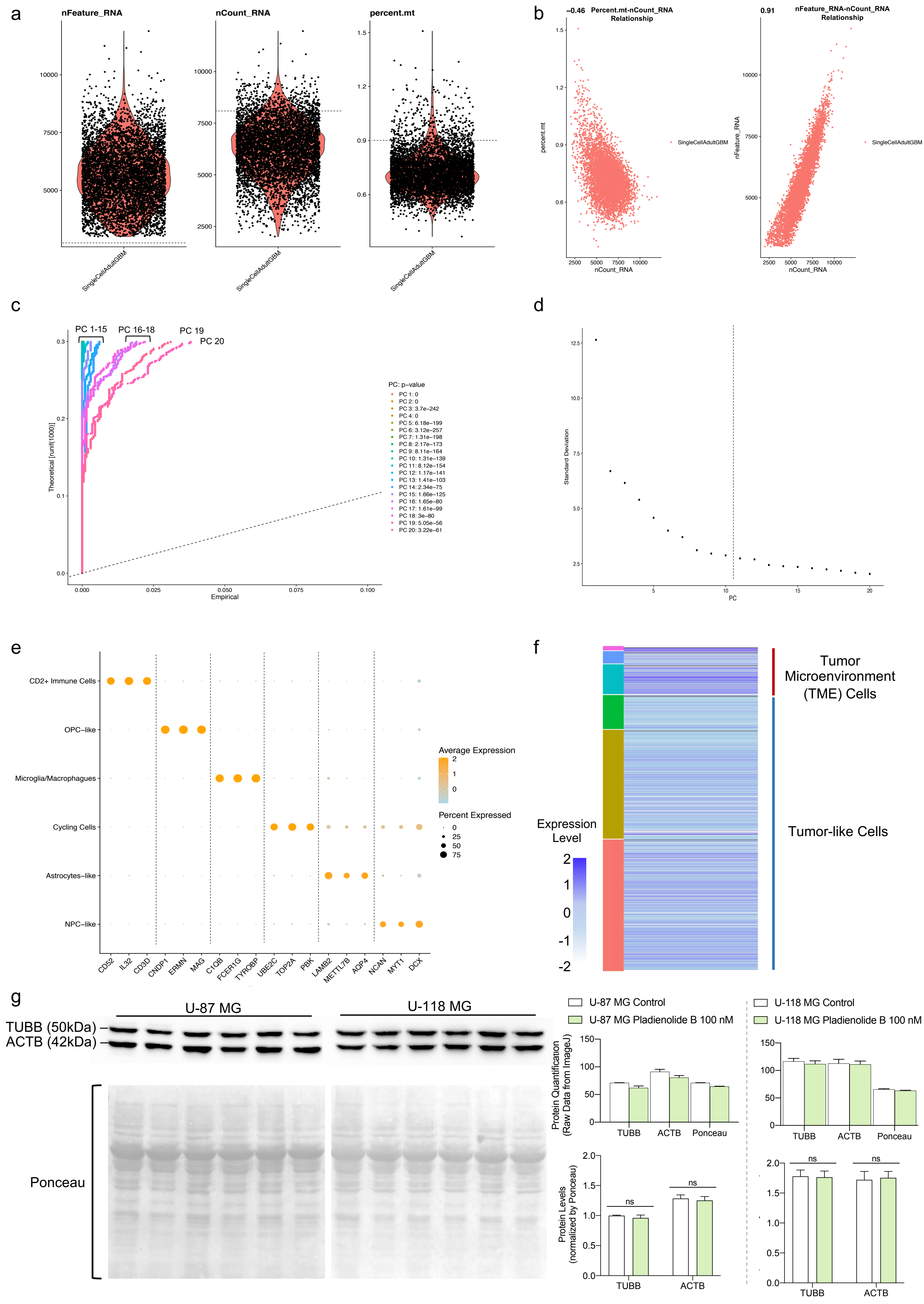
a
nFeature_RNA
nCount_RNA
percent.mt
SingleCellAdultGBM
b
−0.46 Percent.mt-nCount_RNA Relationship
0.91 nFeature_RNA-nCount_RNA Relationship
percent.mt
nFeature_RNA
nCount_RNA
SingleCellAdultGBM
c
PC 1-15
PC 16-18
PC 19
PC 20
Theoretical [runif(1000)]
Empirical
PC: p−value
PC 1: 0
PC 2: 0
PC 3: 3.7e−242
PC 4: 0
PC 5: 6.18e−199
PC 6: 3.12e−257
PC 7: 1.31e−198
PC 8: 2.17e−173
PC 9: 8.11e−164
PC 10: 1.31e−139
PC 11: 8.12e−154
PC 12: 1.17e−141
PC 13: 1.41e−103
PC 14: 2.34e−75
PC 15: 1.66e−125
PC 16: 1.65e−80
PC 17: 1.61e−99
PC 18: 3e−80
PC 19: 5.05e−56
PC 20: 3.22e−61
d
Standard Deviation
PC
e
CD2+ Immune Cells
OPC−like
Microglia/Macrophages
Cycling Cells
Astrocytes−like
NPC−like
CD52
IL32
CD3D
CNDP1
ERMN
MAG
C1QB
FCER1G
TYROBP
UBE2C
TOP2A
PBK
LAMB2
METTL7B
AQP4
NCAN
MYT1
DCX
Average Expression
Percent Expressed
f
Expression Level
Tumor Microenvironment (TME) Cells
Tumor-like Cells
g
U-87 MG
U-118 MG
TUBB (50kDa)
ACTB (42kDa)
Ponceau
U-87 MG Control
U-87 MG Pladienolide B 100 nM
U-118 MG Control
U-118 MG Pladienolide B 100 nM
Protein Quantification (Raw Data from ImageJ)
Protein Levels (normalized by Ponceau)
TUBB
ACTB
Ponceau
ns

Figure S2

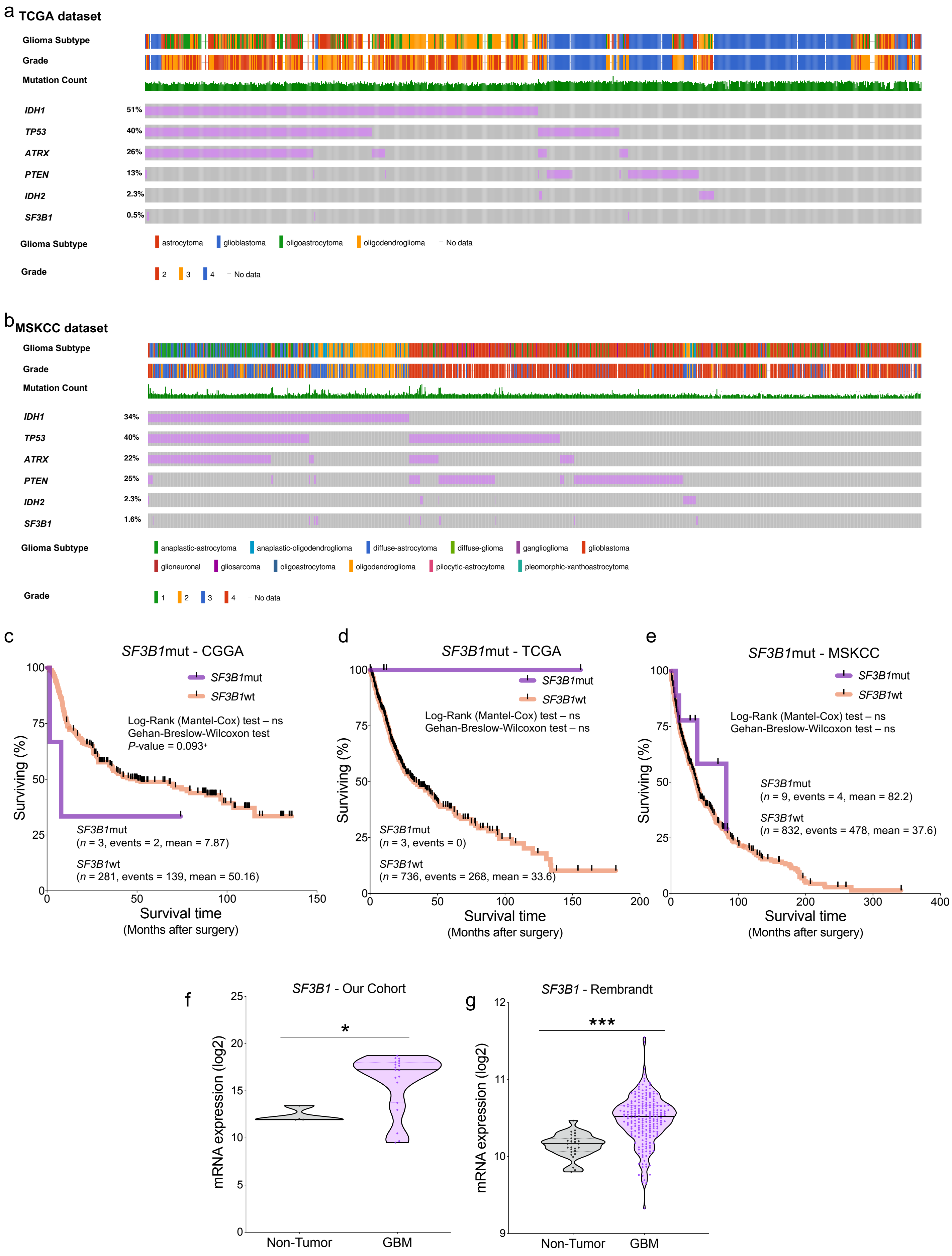

a TCGA dataset
Glioma Subtype
Grade
Mutation Count
IDH1 51%
TP53 40%
ATRX 26%
PTEN 13%
IDH2 2.3%
SF3B1 0.5%
Glioma Subtype
astrocytoma
glioblastoma
oligoastrocytoma
oligodendroglioma
No data
Grade
2
3
4
No data
b MSKCC dataset
Glioma Subtype
Grade
Mutation Count
IDH1 34%
TP53 40%
ATRX 22%
PTEN 25%
IDH2 2.3%
SF3B1 1.6%
Glioma Subtype
anaplastic-astrocytoma
anaplastic-oligodendroglioma
diffuse-astrocytoma
diffuse-glioma
ganglioglioma
glioblastoma
glioneuronal
gliosarcoma
oligoastrocytoma
oligodendroglioma
pilocytic-astrocytoma
pleomorphic-xanthoastrocytoma
Grade
1
2
3
4
No data
c
SF3B1mut - CGGA
SF3B1mut
SF3B1wt
Log-Rank (Mantel-Cox) test – ns
Gehan-Breslow-Wilcoxon test
P-value = 0.093+
SF3B1mut
(n = 3, events = 2, mean = 7.87)
SF3B1wt
(n = 281, events = 139, mean = 50.16)
Surviving (%)
Survival time
(Months after surgery)
d
SF3B1mut - TCGA
SF3B1mut
SF3B1wt
Log-Rank (Mantel-Cox) test – ns
Gehan-Breslow-Wilcoxon test – ns
SF3B1mut
(n = 3, events = 0)
SF3B1wt
(n = 736, events = 268, mean = 33.6)
Surviving (%)
Survival time
(Months after surgery)
e
SF3B1mut - MSKCC
SF3B1mut
SF3B1wt
Log-Rank (Mantel-Cox) test – ns
Gehan-Breslow-Wilcoxon test – ns
SF3B1mut
(n = 9, events = 4, mean = 82.2)
SF3B1wt
(n = 832, events = 478, mean = 37.6)
Surviving (%)
Survival time
(Months after surgery)
f
SF3B1 - Our Cohort
*
mRNA expression (log2)
Non-Tumor
GBM
g
SF3B1 - Rembrandt
***
mRNA expression (log2)
Non-Tumor
GBM

Figure S3

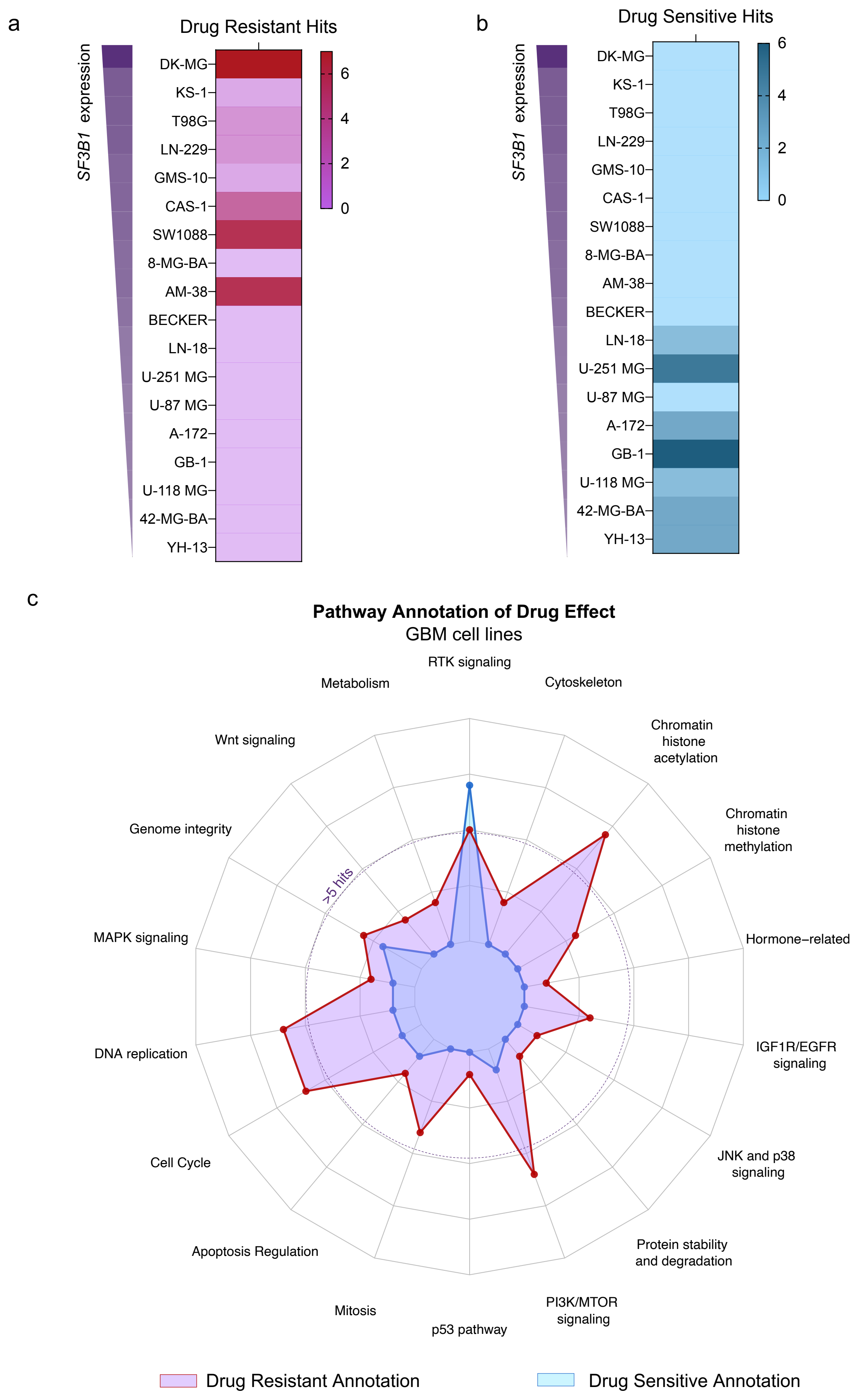
a
Drug Resistant Hits
SF3B1 expression
DK-MG
KS-1
T98G
LN-229
GMS-10
CAS-1
SW1088
8-MG-BA
AM-38
BECKER
LN-18
U-251 MG
U-87 MG
A-172
GB-1
U-118 MG
42-MG-BA
YH-13
6
4
2
0
b
Drug Sensitive Hits
SF3B1 expression
DK-MG
KS-1
T98G
LN-229
GMS-10
CAS-1
SW1088
8-MG-BA
AM-38
BECKER
LN-18
U-251 MG
U-87 MG
A-172
GB-1
U-118 MG
42-MG-BA
YH-13
6
4
2
0
c
Pathway Annotation of Drug Effect
GBM cell lines
RTK signaling
Cytoskeleton
Chromatin histone acetylation
Chromatin histone methylation
Hormone−related
IGF1R/EGFR signaling
JNK and p38 signaling
Protein stability and degradation
PI3K/MTOR signaling
p53 pathway
Mitosis
Apoptosis Regulation
Cell Cycle
DNA replication
MAPK signaling
Genome integrity
Wnt signaling
Metabolism
>5 hits
Drug Resistant Annotation
Drug Sensitive Annotation

Figure S4

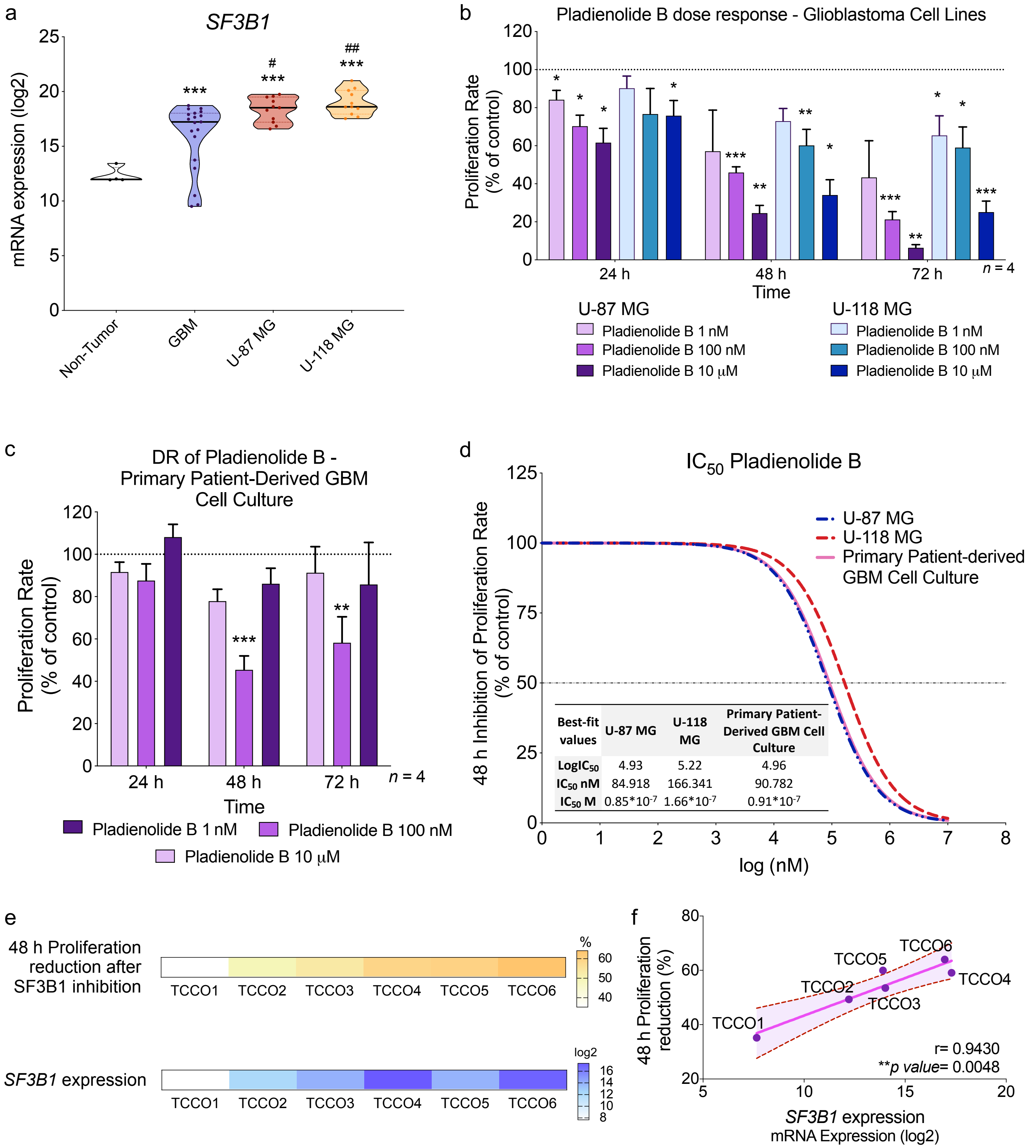

a
SF3B1
mRNA expression (log2)
25
20
15
10
5
0
***
#
***
##
***
Non-Tumor
GBM
U-87 MG
U-118 MG
b
Pladienolide B dose response - Glioblastoma Cell Lines
Proliferation Rate
(% of control)
120
100
80
60
40
20
0
24 h
48 h
72 h
Time
n = 4
U-87 MG
Pladienolide B 1 nM
Pladienolide B 100 nM
Pladienolide B 10 μM
U-118 MG
Pladienolide B 1 nM
Pladienolide B 100 nM
Pladienolide B 10 μM
c
DR of Pladienolide B -
Primary Patient-Derived GBM
Cell Culture
Proliferation Rate
(% of control)
24 h
48 h
72 h
Time
n = 4
Pladienolide B 1 nM
Pladienolide B 100 nM
Pladienolide B 10 μM
d
IC50 Pladienolide B
48 h Inhibition of Proliferation Rate
(% of control)
125
100
75
50
25
0
U-87 MG
U-118 MG
Primary Patient-derived
GBM Cell Culture
Best-fit values | U-87 MG | U-118 MG | Primary Patient-Derived GBM Cell Culture
LogIC50 | 4.93 | 5.22 | 4.96
IC50 nM | 84.918 | 166.341 | 90.782
IC50 M | 0.85*10-7 | 1.66*10-7 | 0.91*10-7
log (nM)
e
48 h Proliferation
reduction after
SF3B1 inhibition
TCCO1
TCCO2
TCCO3
TCCO4
TCCO5
TCCO6
%
60
50
40
SF3B1 expression
log2
16
14
12
10
8
f
48 h Proliferation
reduction (%)
80
60
40
20
TCCO1
TCCO2
TCCO3
TCCO4
TCCO5
TCCO6
r= 0.9430
**p value= 0.0048
SF3B1 expression
mRNA Expression (log2)

Figure S5

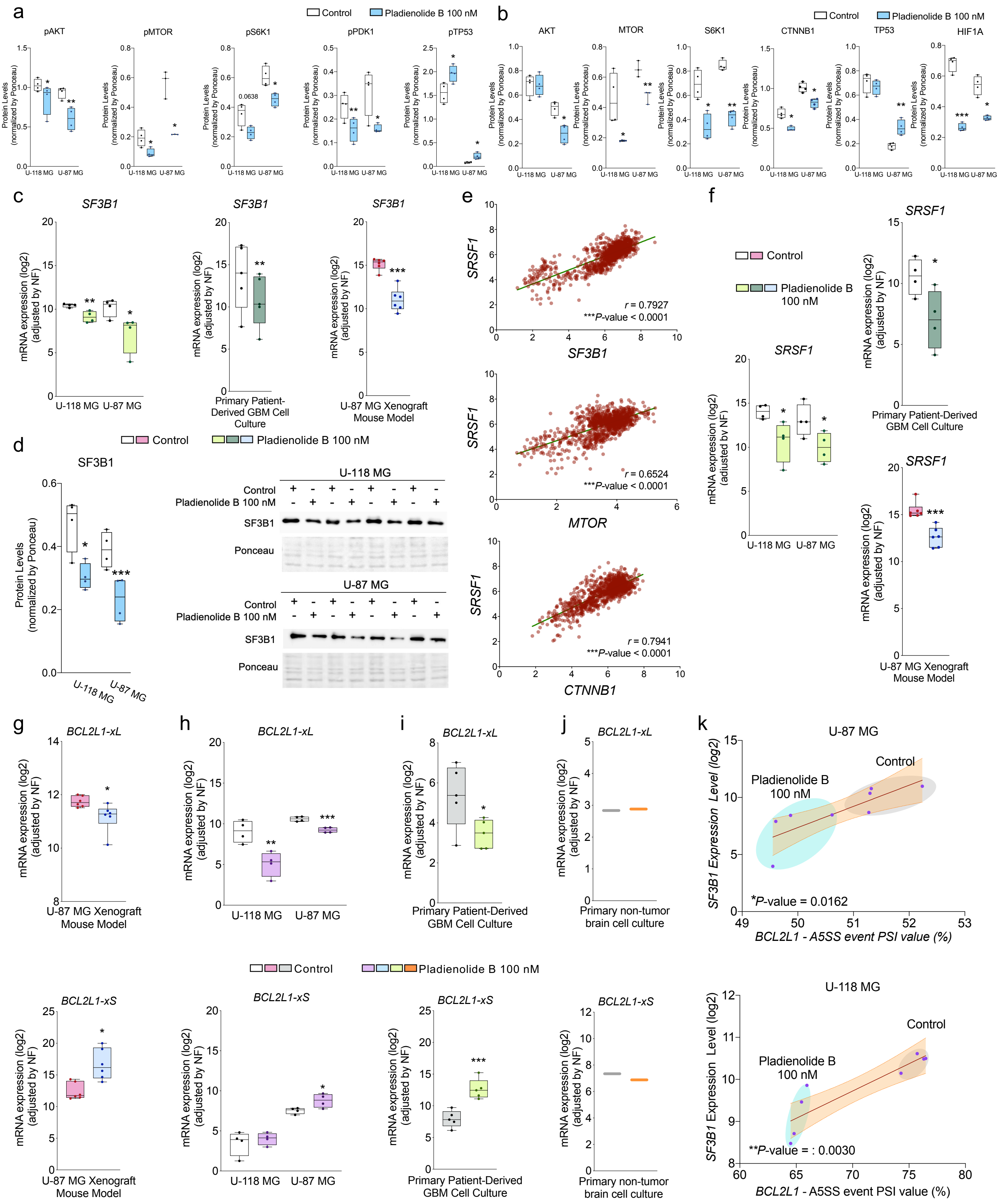

a
Control
Pladienolide B 100 nM
pAKT
pMTOR
pS6K1
pPDK1
pTP53
Protein Levels (normalized by Ponceau)
U-118 MG
U-87 MG
0.0638
b
AKT
MTOR
S6K1
CTNNB1
TP53
HIF1A
c
SF3B1
mRNA expression (log2) (adjusted by NF)
Primary Patient-Derived GBM Cell Culture
U-87 MG Xenograft Mouse Model
d
SF3B1
Pladienolide B 100 nM
Ponceau
e
SRSF1
r = 0.7927
***P-value < 0.0001
MTOR
r = 0.6524
CTNNB1
r = 0.7941
f
SRSF1
g
h
i
j
BCL2L1-xL
BCL2L1-xS
Primary non-tumor brain cell culture
k
U-87 MG
U-118 MG
SF3B1 Expression Level (log2)
BCL2L1 - A5SS event PSI value (%)
*P-value = 0.0162
**P-value = : 0.0030

Table S1

Parameters	Glioma patients
CGGA WEseq	
**Patients (*n*)**	284
**Gender (M/F)**	168 (59.15 %) / 116 (40.85 %)
**Primary LGG**	124 (43.67 %)
**Recurrent LGG**	58 (20.42 %)
**Primary GBM**	54 (19.01 %)
**Recurrent GBM**	48 (16.90 %)
**Age at surgical intervention (mean±desvest)**	41.97 ± 12.34
**Grade II**	91 (32.04 %)
**Grade III**	91 (32.04 %)
**Grade IV**	102 (35.92 %)
**GBM *IDH1* status (wt/mut)**	151 (53.17 %) / 133 (46.83 %)
**GBM survival rate (Mean - Months)**	42.03 ± 34.89
TCGA	
**Patients (*n*)**	746
**Gender (M/F)**	441 (59.11 %) / 305 (40.88 %)
**Primary LGG**	451 (60.46 %)
**Recurrent LGG**	N/A
**Primary GBM**	295 (39.54 %)
**Recurrent GBM**	N/A
**Age at surgical intervention (mean±desvest)**	50.26 ± 15.91
**Grade II**	212 (28.42 %)
**Grade III**	239 (32.04 %)
**Grade IV**	295 (39.54 %)
**GBM *IDH1* status (wt/mut)**	315 (50.97 %) / 303 (49.03 %)
**GBM survival rate (Mean - Months)**	19.08 ± 24.57
MSKCC	
**Patients (*n*)**	923
**Gender (M/F)**	557 (60.35 %) / 366 (39.65 %)
**Primary LGG**	273 (29.58 %)
**Recurrent LGG**	123 (13.33 %)
**Primary GBM**	431 (46.70 %)
**Recurrent GBM**	118 (12.78 %)
**Age at surgical intervention (mean±desvest)**	49.49 ± 16.06
**Grade II**	145 (15.71 %)
**Grade III**	251 (27.19 %)
**Grade IV**	549 (59.48 %)
**GBM *IDH1* status (wt/mut)**	584 (63.27 %) /339 (33.8 %)
**GBM survival rate (Mean - Months)**	40.61 ± 48.49

Table S2

Parameters	Control patients	GBM patients
Our Cohort		
**Patients (*n*)**	4	22
**Gender (M/F)**	1(25 %) / 3(75 %)	17(77.27%) / 5(23.73%)
**Age at surgical intervention (mean±desvest)**	51.2 ± 5.38	59.81 ± 12.77
**Grade IV**	-	22
**%KI67 (mean±desvest)**	-	33.9 ± 14.76
**% of P53 positive**	-	72.73 %
**% of PAGF positive**	-	72.73 %
**% of *IDH1* positive**	-	18.18 %
**GBM survival rate (Mean - Months)**	-	14.92
Rembrandt		
**Patients (*n*)**	28	219
**Gender (M/F)**	N/A	N/A
**Age at surgical intervention (mean±desvest)**	N/A	N/A
**Grade IV**	-	219
**Molecular subtype:Classical**	-	99 (45.21 %)
**Molecular subtype:Mesenchymal**	-	37 (16.89 %)
**Molecular subtype:Proneural**	-	39 (17.81 %)
**G-CIMP status (Positive/Negative)**	-	11 (5.02 %) / 208 (94.98 %)
**GBM survival rate (Mean - Months)**	-	19.83
CGGA		
**Patients (*n*)**	-	388
**Gender (M/F)**	-	236 (60.82 %) / 152 (29.18 %)
**Age at surgical intervention (mean±desvest)**	-	48.01 ± 13.30
**Grade IV**	-	388
**Molecular GBM subtype:Classical**	-	107 (25.58 %)
**Molecular GBM subtype:Mesenchymal**	-	89 (30.90 %)
**Molecular GBM subtype:Proneural**	-	83 (21.39 %)
**Primary GBM**	-	225 (57.99 %)
**Secondary GBM**	-	30 (6.15 %)
**Recurrent GBM**	-	133 (35.86 %)
**GBM *IDH1* status (wt/mut)**		288 (74.23 %) / 100 (25.77 %)
**GBM survival rate (Mean - Months)**	-	17.15

Table S3

P-value	pct.1	pct.2	p_val_adj	cluster	gene	
0	0.819	0.429	0	0	*DCX*	**NPC-like**
0	0.612	0.132	0	0	*MYT1*	
8.55E-276	0.675	0.183	1.87E-270	0	*NCAN*	
3.22E-267	0.61	0.159	7.04E-263	0	*MIAT*	
9.94E-261	0.845	0.611	2.17E-256	0	*CD24*	
1.10E-253	0.565	0.126	2.40E-249	0	*ELAVL4*	
4.36E-220	0.583	0.175	9.53E-216	0	*TAGLN3*	
1.08E-217	0.415	0.055	2.35E-213	0	*STMN2*	
2.16E-200	0.606	0.209	4.73E-196	0	*B4GALNT1*	
6.80E-138	0.363	0.079	1.49E-132	0	*NNAT*	
0	0.733	0.147	0	1	*AQP4*	**Astrocytes-like**
0	0.645	0.126	0	1	*METTL7B*	
0	0.85	0.246	0	1	*LAMB2*	
0	0.856	0.331	0	1	*TNC*	
0	0.62	0.121	0	1	*EMP1*	
2.66E-289	0.716	0.186	5.82E-285	1	*HOPX*	
9.51E-288	0.811	0.262	2.08E-282	1	*S100A16*	
3.06E-274	0.813	0.27	6.69E-270	1	*LGALS3*	
2.82E-256	0.844	0.488	6.17E-252	1	*F3*	
3.09E-183	0.387	0.063	6.76E-179	1	*CHI3L1*	
0	0.836	0.044	0	2	*PBK*	**Cylcing Cells**
0	0.887	0.073	0	2	*TOP2A*	
0	0.808	0.071	0	2	*UBE2C*	
0	0.882	0.073	0	2	*BIRC5*	
0	0.895	0.102	0	2	*UBE2T*	
0	0.753	0.054	0	2	*FAM64A*	
0	0.863	0.078	0	2	*TPX2*	
0	0.886	0.112	0	2	*NUSAP1*	
0	0.803	0.07	0	2	*CDK1*	
0	0.799	0.044	0	2	*NUF2*	
0	0.983	0.007	0	3	*TYROBP*	**Microglia/Macrophagues**
0	0.977	0.018	0	3	*FCER1G*	
0	0.924	0.008	0	3	*C1QB*	
0	0.968	0.031	0	3	*RGS1*	
0	0.971	0.026	0	3	*SRGN*	
0	0.947	0.005	0	3	*C1QA*	
0	0.935	0.007	0	3	*C1QC*	
0	0.947	0.006	0	3	*FCGR3A*	
0	0.996	0.031	0	3	*LAPTM5*	
0	0.996	0.071	0	3	*HLA.DRA*	
0	0.99	0.009	0	4	*MAG*	**OPC-like**
0	0.995	0.022	0	4	*ERMN*	
0	0.97	0.013	0	4	*CNDP1*	
0	1	0.031	0	4	*ENPP2*	
0	0.98	0.011	0	4	*KLK6*	
0	0.98	0.013	0	4	*SPOCK3*	
0	1	0.074	0	4	*PTGDS*	
0	1	0.091	0	4	*TF*	
0	0.985	0.043	0	4	*RNASE1*	
0	1	0.08	0	4	*TMEM144*	
0	0.91	0.001	0	5	*CD3D*	**CD2+ Immune Cells**
0	0.955	0.021	0	5	*IL32*	
0	0.866	0.006	0	5	*CD52*	
0	0.866	0.003	0	5	*CD2*	
0	0.97	0.002	0	5	*CD3E*	
0	0.866	0.019	0	5	*IL2RG*	
2.33E-230	0.896	0.038	5.10E-225	5	*CYTIP*	
1.74E-186	0.881	0.048	3.80E-182	5	*PTPRCAP*	
8.28E-180	0.761	0.035	1.81E-175	5	*IFITM1*	
2.03E-141	1	0.09	4.43E-137	5	*LCP1*	

Table S4

Parameters	Non-tumor patients	GBMs patients
CPTAC		
**Patients (*n*)**	10	100
**Gender (M/F)**	5(50 %) / 5(50 %)	56(56 %) / 44(44 %)
**Age at surgical intervention (mean±desvest)**	57.5 ± 9.10	57.93 ± 12.44
**Grade IV**	-	100
**GBM survival rate (Mean - Months)**	-	11.26

Table S5

#term ID	term description	observed gene count	background gene count	*P-value* FDR <0.01
HSA-8953854	Metabolism of RNA	126	652	1.68E-28
HSA-72172	mRNA Splicing	66	186	2.64E-26
HSA-72203	Processing of Capped Intron-Containing Pre-mRNA	73	234	2.64E-26
HSA-72163	mRNA Splicing - Major Pathway	64	178	5.75E-26
HSA-74160	Gene expression (Transcription)	143	1366	6.95E-11
HSA-73857	RNA Polymerase II Transcription	125	1233	1.52E-08
HSA-392499	Metabolism of proteins	166	1948	1.65E-06
HSA-72187	mRNA 3'-end processing	18	56	3.52E-06
HSA-109688	Cleavage of Growing Transcript in the Termination Region	19	65	4.60E-06
HSA-162599	Late Phase of HIV Life Cycle	27	135	5.92E-06
HSA-3700989	Transcriptional Regulation by TP53	48	359	6.39E-06
HSA-162587	HIV Life Cycle	28	148	7.95E-06
HSA-162906	HIV Infection	35	224	1.29E-05
HSA-1640170	Cell Cycle	65	586	1.59E-05
HSA-674695	RNA Polymerase II Pre-transcription Events	20	84	1.98E-05
HSA-69620	Cell Cycle Checkpoints	38	265	2.24E-05
HSA-72202	Transport of Mature Transcript to Cytoplasm	19	77	2.24E-05
HSA-5696398	Nucleotide Excision Repair	22	109	5.00E-05
HSA-112382	Formation of RNA Pol II elongation complex	16	61	7.93E-05
HSA-159236	Transport of Mature mRNA derived from an Intron-Containing Transcript	17	69	7.93E-05
HSA-6781823	Formation of TC-NER Pre-Incision Complex	15	53	7.93E-05
HSA-73894	DNA Repair	38	290	0.00011
HSA-167172	Transcription of the HIV genome	17	73	0.00013
HSA-68886	M Phase	42	343	0.00015
HSA-212436	Generic Transcription Pathway	98	1112	0.00017
HSA-68882	Mitotic Anaphase	28	186	0.00021
HSA-72165	mRNA Splicing - Minor Pathway	14	52	0.00021
HSA-199991	Membrane Trafficking	62	612	0.00022
HSA-2467813	Separation of Sister Chromatids	27	178	0.00024
HSA-597592	Post-translational protein modification	114	1366	0.00024
HSA-167152	Formation of HIV elongation complex in the absence of HIV Tat	13	48	0.00035
HSA-5663205	Infectious disease	42	363	0.00039
HSA-1632852	Macroautophagy	15	66	0.00043
HSA-5653656	Vesicle-mediated transport	63	649	0.00053
HSA-69618	Mitotic Spindle Checkpoint	19	105	0.00054
HSA-380972	Energy dependent regulation of mTOR by LKB1-AMPK	10	29	0.00058
HSA-6781827	Transcription-Coupled Nucleotide Excision Repair (TC-NER)	16	78	0.00062
HSA-69278	Cell Cycle Mitotic	50	483	0.00080
HSA-983168	Antigen processing: Ubiquitination & Proteasome degradation	36	303	0.00082
HSA-8953897	Cellular responses to external stimuli	48	459	0.00087
HSA-165159	mTOR signalling	11	39	0.00094
HSA-5633007	Regulation of TP53 Activity	23	159	0.0015
HSA-5696395	Formation of Incision Complex in GG-NER	11	42	0.0016
HSA-3108232	SUMO E3 ligases SUMOylate target proteins	22	154	0.0025
HSA-167200	Formation of HIV-1 elongation complex containing HIV-1 Tat	11	46	0.0031
HSA-5696399	Global Genome Nucleotide Excision Repair (GG-NER)	15	83	0.0031
HSA-6782210	Gap-filling DNA repair synthesis and ligation in TC-NER	13	64	0.0031
HSA-6782135	Dual incision in TC-NER	13	65	0.0034
HSA-983169	Class I MHC mediated antigen processing & presentation	38	365	0.0048
HSA-141430	Inactivation of APC/C via direct inhibition of the APC/C complex	7	19	0.0052
HSA-68877	Mitotic Prometaphase	24	190	0.0054
HSA-6807070	PTEN Regulation	19	135	0.0068
HSA-6804756	Regulation of TP53 Activity through Phosphorylation	15	92	0.0070
HSA-6807505	RNA polymerase II transcribes snRNA genes	13	74	0.0089
HSA-72086	mRNA Capping	8	29	0.0089
HSA-429914	Deadenylation-dependent mRNA decay	11	55	0.0090
HSA-72766	Translation	31	288	0.0090
HSA-166208	mTORC1-mediated signalling	7	22	0.0092
HSA-6796648	TP53 Regulates Transcription of DNA Repair Genes	12	65	0.0092
HSA-3214847	HATs acetylate histones	16	107	0.0095
HSA-167161	HIV Transcription Initiation	10	47	0.0097
HSA-167162	RNA Polymerase II HIV Promoter Escape	10	47	0.0097
HSA-176408	Regulation of APC/C activators between G1/S and early anaphase	13	76	0.0097
HSA-69052	Switching of origins to a post-replicative state	14	86	0.0097
HSA-73776	RNA Polymerase II Promoter Escape	10	47	0.0097
HSA-73779	RNA Polymerase II Transcription Pre-Initiation And Promoter Opening	10	47	0.0097
HSA-75953	RNA Polymerase II Transcription Initiation	10	47	0.0097

Table S6

Gene	Accession Number	Sense Primer Sequence	AntiSnese Primer Sequence	Product Size
ACTB	NM_001101	ACTCTTCCAGCCTTCCTTCCT	CAGTGATCTCCTTCTGCATCCT	176
GAPDH	NM_002046	AATCCCATCACCATCTTCCA	AAATGAGCCCCAGCCTTC	122
HPRT	NM_000194.2	CTGAGGATTTGGAAAGGGTGT	TAATCCAGCAGGTCAGCAAAG	157
SF3B1	NM_012433.3	CAGTTCCGTCTGTGTGTTCG	GCTGCCTTCTTGCCTTGA	101
VEGFA	NM_001171623.1	GGGCAGAATCATCACGAAGT	ATCTGCATGGTGATGTTGGA	218
MKI67	NM_002417.5	GACATCCGTATCCAGCTTCCT	GCCGTACAGGCTCATCAATAAC	139
EGFR	NM_005228.5	CCTTGCCGCAAAGTGTGTA	TGTGAAGGAGTCACCCCTAAAT	153
CDK4	NM_000075.4	ACAGTTCGTGAGGTGGCTTT	TACCTTGATCTCCCGGTCAG	111
PDGFRA	NM_006206.6	CAGGCCCCATTTACATCATC	CATAGCTCCGTGTGCTTTCA	162
PDGFRB	NM_002609.4	AGCTTGTCCTCAATGTCTCCA	GGGATCTGGCACAAAGATGTA	248
SRSF3	NM_003017.4	TAACCCTAGATCTCGAAATGCATC	CATAGTAGCCAAAAGCCCGTT	117
RBM22	NM_018047.2	CTCTGGGTTCCAACACCTACA	GGCACAGATTTTGCATTCCT	137
PTBP1	NM_002819.4	TGGGTCGGTTCCTGCTATT	CAGATCCCCGCTTTGTAC	111
RBM3	NM_006743.4	AAGCTCTTCGTGGGAGGG	TTGACAACGACCACCTCAGA	98
MYC	NM_002467.6	CTCGGATTCTCTGCTCTCCTC	TTCCTCATCTTCTTGTTCCTCCT	124
CCND1	NM_053056.3	CCTCGGTGTCCTACTTCAAAT	TCCTCCTCGCACTTCTGTTC	109
SRSF1	NM_006924.5	TGTCTCTGGACTGCCTCCA	TGCCATCTCGGTAAACATCA	98
BCL2L1	NM_138578.3	TCCCCATGGCAGCAGTAAAG	GGTGGGAGGGTAGAGTGGAT	494 *BCL2L1-xS*<br>305 *BCL2L1-xL*
BCL2-xS	NM_001191.4	GAGCTTTGAACAGGATACTTTTGTG	GAAGAGTGAGCCCAGCAGAA	97
BCL2-xL	NM_001317919.2<br>NM_001317920.2<br>NM_001317921.2<br>NM_001322239.2<br>NM_001322240.2<br>NM_001322242.2<br>NM_138578.3	GATGGCCACTTACCTGAATGA	TGCTGCATTGTTCCCATAGA	94
KLF6	NM_001300.6<br>NM_001160124.2<br>NM_001160125.2	CATCTTCTCCGGAACTGAGC	GCTATGCCGCTTCTTACAGG	585 KLF6a<br>459 KLF6b<br>461 KLF6c
CRK	NM_005206.5<br>NM_016823.4	GCAAGAGAGGGATGATTCCA	ATGGGAAGTGACCTCGTTTG	333 CRKsvI<br>163 CRKsvII
MCL1	NM_182763.3<br>NM_021960.5	AGACCTTACGACGGGTTGG	ACCAGCTCCTACTCCAGCAA	401 MCL1L<br>153 MCL1S
CASP2	NM_001224.5<br>NM_032982.4	AACTGCCCAAGCCTACAGAA	CTGCGTGGTTCTTTCCATCT	167 CASP2S<br>106 CASP2L
RAC1	NM_018890.4<br>NM_006908.5	CCCTATCCTATCCGCAAACA	GGACTCACAAGGGAAAAGCA	55 RAC1<br>112 RAC1b
BIRC5	NM_001012270.2<br>NM_001012271.2<br>NM_001168.3	CCACTGAGAACGAGCCAGA	GAGAGAAGCAGCCACTGTTACC	516 BIRC5<br>585 BIRC5-2b<br>398 BIRC5-ΔEx3
MST1R	NM_002447.4<br>XM_011533739.2	GATGGAGCTGCTGGCTTTAC	ATCTACGCAGACCTGCAATG	270 RON<br>150 RONlack11
SPP1	NM_001251830.2<br>NM_001040058.2<br>NM_000582.3<br>NM_001040060.2	GCATCACCTGTGCCATACC	TGGACTTACTTGGAAGGGTCTC	390 SPP1<br>201 SPP1a<br>159 SPP1b<br>120 SPP1c

Table S7

Name	3'-5' ASO sequence	5'-3' *BCL2L1* Targeted Region	Oligo Size
ASO1_BCL2L1	**TGTT**CAAAGCTCTGATATGC**TGTC**	GACAGCATATCAGAGCTTTGAACA	24 bases
ASO2_BCL2L1	**CCCC**ATCCCGGAAGA**GTTC**	GAACTCTTCCGGGATGGGG	19 bases
ASO3_BCL2L1	**ACAA**TGCGACCCCAG**TTTA**	TAAACTGGGGTCGCATTGT	19 bases
ASO4_BCL2L1	**CGCC**GAAGGAGAAAAA**GGCC**	GGCCTTTTTCTCCTTCGGCG	20 bases
ASO5_BCL2L1	**TTTC**CACGCACAGT**GCCC**	GGGCACTGTGCGTGGAAA	18 bases

Table S8

Drug Name	Drug Target	Target Pathway Annotation
Wnt-C59	PORCN	WNT signaling
IWP-2	PORCN	WNT signaling
Staurosporine	Broad spectrum kinase inhibitor	RTK signaling
GSK1904529A	IGF1R, IR	RTK signaling
Crizotinib	MET, ALK, ROS1	RTK signaling
Foretinib	MET, KDR, TIE2, VEGFR3/FLT4, RON, PDGFR, FGFR1, EGFR	RTK signaling
Cediranib	VEGFR, FLT1, FLT2, FLT3, FLT4, KIT, PDGFRB	RTK signaling
ML323	USP1, UAF1	Protein stability and degradation
MK-2206	AKT1, AKT2	PI3K/MTOR signaling
Rapamycin	MTORC1	PI3K/MTOR signaling
Pilaralisib	PI3K	PI3K/MTOR signaling
Dactolisib	PI3K (class 1), MTORC1, MTORC2	PI3K/MTOR signaling
Alpelisib	PI3Kalpha	PI3K/MTOR signaling
CZC24832	PI3Kgamma	PI3K/MTOR signaling
PRIMA-1MET	TP53 activation	p53 pathway
Vinorelbine	Microtubule destabiliser	Mitosis
Docetaxel	Microtubule stabiliser	Mitosis
Paclitaxel	Microtubule stabiliser	Mitosis
TTK_3146	TTK	Mitosis
AICA Ribonucleotide	AMPK agonist	Metabolism
GSK2606414	PERK	Metabolism
TAK-715	p38alpha, p38beta	JNK and p38 signaling
Linsitinib	IGF1R	IGF1R signaling
NVP-ADW742	IGF1R	IGF1R signaling
Fulvestrant	ESR	Hormone-related
VE-822	ATR	Genome integrity
Mirin	MRE11	Genome integrity
BIBR-1532	TERT	Genome integrity
VX-11e	ERK2	ERK MAPK signaling
KRAS (G12C) Inhibitor-12	KRAS (G12C)	ERK MAPK signaling
Lapatinib	EGFR, ERBB2	EGFR signaling
Cyclophosphamide	Alkylating agent	DNA replication
Doxorubicin	Anthracycline	DNA replication
Mitomycin-C	DNA crosslinker	DNA replication
Pyridostatin	G-quadruplex stabiliser	DNA replication
Etoposide	TOP2	DNA replication
Carmustine		DNA replication
BDP-00009066	MRCKB_HUMAN	Cytoskeleton
PAK_5339	PAK1, PAK2	Cytoskeleton
I-BET-762	BRD2, BRD3, BRD4	Chromatin other
JQ1	BRD2, BRD3, BRD4, BRDT	Chromatin other
PFI-1	BRD4	Chromatin other
PFI3	Polybromo 1, SMARCA4, SMARCA2	Chromatin other
EPZ5676	DOT1L	Chromatin histone methylation
GSK343	EZH2	Chromatin histone methylation
GSK591	PMRT5	Chromatin histone methylation
OF-1	BRPF1B, BRPF2	Chromatin histone acetylation
Vorinostat	HDAC inhibitor Class I, IIa, IIb, IV	Chromatin histone acetylation
Dacinostat	HDAC1	Chromatin histone acetylation
JQ12	HDAC1, HDAC2	Chromatin histone acetylation
Entinostat	HDAC1, HDAC3	Chromatin histone acetylation
ACY-1215	HDAC6	Chromatin histone acetylation
PCI-34051	HDAC8, HDAC6, HDAC1	Chromatin histone acetylation
Flavopiridol	CDK	Cell cycle
RO-3306	CDK1	Cell cycle
AZD5438	CDK2	Cell cycle
Palbociclib	CDK4, CDK6	Cell cycle
THZ-2-102-1	CDK7	Cell cycle
Wee1 Inhibitor	WEE1, CHEK1	Cell cycle
Embelin	XIAP	Apoptosis regulation
AZD5582	XIAP, cIAP	Apoptosis regulation

Table S9

Drug Name	Drug Target	Target Pathway Annotation
Staurosporine	Broad spectrum kinase inhibitor	RTK signaling
Motesanib	VEGFR, RET, KIT, PDGFR	RTK signaling
SB505124	TGFBR1, ACVR1B, ACVR1C	RTK signaling
PD173074	FGFR1, FGFR2, FGFR3	RTK signaling
Tivozanib	VEGFR1, VEGFR2, VEGFR3	RTK signaling
Pazopanib	CSF1R, KIT, PDGFRA, PDGFRB	RTK signaling
PD173074	FGFR1, FGFR2, FGFR3	RTK signaling
